# Only kosmotrope anions trigger fibrillization of the recombinant core spidroin eADF4(C16) from *Araneus diadematus*

**DOI:** 10.1002/pro.4832

**Published:** 2023-12-01

**Authors:** Veronika Hovanová, Andrej Hovan, Martin Humenik, Erik Sedlák

**Affiliations:** ^1^ Center for Interdisciplinary Biosciences, Technology and Innovation Park P.J. Šafárik University Košice Slovakia; ^2^ Department of Biophysics, Faculty of Science P.J. Šafárik University Košice Slovakia; ^3^ Department of Biomaterials, Faculty of Engineering Science University Bayreuth Bayreuth Germany; ^4^ Department of Biochemistry, Faculty of Science P.J. Šafárik University Košice Slovakia

**Keywords:** fibrils, Hofmeister anions, recombinant protein, self‐assembly, spider silk

## Abstract

Recombinant core spidroin eADF4(C16) has received increasing attention due to its ability to form micro‐ and nano‐structured scaffolds, which are based on nanofibrils with great potential for biomedical and biotechnological applications. Phosphate anions have been demonstrated to trigger the eADF4(C16) self‐assembly into cross‐beta fibrils. In the present work, we systematically addressed the effect of nine sodium anions, namely SO_4_^2−^, HPO_4_^2−^ (Pi), F^−^, Cl^−^, Br^−^, NO_3_^−^, I^−^, SCN^−^, and ClO_4_^−^ from the Hofmeister series on the in vitro self‐assembly kinetics of eADF4(C16). We show that besides the phosphate anions, only kosmotropic anions such as sulfate and fluoride can initiate the eADF4(C16) fibril formation. Global analysis of the self‐assembly kinetics, utilizing the platform AmyloFit, showed the nucleation‐based mechanism with a major role of secondary nucleation, surprisingly independent of the type of the kosmotropic anion. The rate constant of the fibril elongation in mixtures of phosphate anions with other studied anions correlated with their kosmotropic or chaotropic position in the Hofmeister series. Our findings suggest an important role of anion hydration in the eADF4(C16) fibrillization process.

## 1 INTRODUCTION

The bottom‐up assembly is a promising nature‐inspired strategy for designing functional biomaterials (Gaspar et al., 2020; Weisenberger & Deans, 2018). The building blocks of these materials are arranged in a bottom‐up manner into complex nano‐ to macroscale hierarchical architectures, which allow for preparation of sustainable, multifunctional materials with unique combinations of strength and toughness (Lendel & Solin, 2021; Ling et al., 2018; Lombardo et al., 2020; Olson et al., 2021; Percebom et al., 2018; Shen et al., 2021; Wegst et al., 2015). There is a growing focus on proteinaceous nanofibrils made of recombinant spider silk proteins or silk fibroin from *Bombyx mori*, which can be manufactured into gels and intensively applied in 3D printing and tissue engineering techniques (de Oliveira et al., 2023; Humenik et al., 2014; Kamada et al., 2023; Kim et al., 2021; Lamberger et al., 2022; Schacht et al., 2015; Scheibel et al., 2022; Steiner et al., 2021; Sun & Marelli, 2020).

The recombinant spider silk protein, eADF4(C16), possesses the ability to form diverse nanostructures based on nanofibrils. The protein structure mimics the core domain of fibroin 4 from the dragline silk of the European garden spider *Araneus diadematus*, containing a 16‐times repeating C‐module that includes polyalanine and glycine‐proline‐rich motives (Huemmerich et al., 2004; Humenik et al., 2014; Rammensee et al., 2008; Slotta et al., 2007). The eADF4(C16) is already known for its remarkable mechanical properties, biocompatibility, biodegradability, and low immunogenicity, making it an interesting subject of study (Humenik et al., 2011, 2021; Leal‐Egana & Scheibel, 2010; Muller‐Herrmann & Scheibel, 2015; Steiner et al., 2019; Trossmann & Scheibel, 2023). In contrast to numerous other proteins, which form fibrils under harsh denaturing conditions, such as high temperature, acidic pH, and/or vigorous shaking (Ponikova et al., 2015; Rahamtullah & Mishra, 2021; Raman et al., 2005; Su & Chang, 2001), the eADF4(C16) self‐assembles into nanofibrils in the presence of <300 mM phosphate ions (Pi) under quiescent conditions and at room temperature (Humenik et al., 2014). It has been shown that phosphates, and with slightly lower efficiency, also sulfates induce eADF4(C16) fibril formation, suggesting an important role of the kosmotropic nature of the anions in the self‐assembly process (Collins, 2004; Kang et al., 2020). Nevertheless, the impact of kosmotropic and chaotropic anions from the Hofmeister series on the transition of the protein into insoluble fibrils has not been systematically addressed yet.

In 1888, Franz Hofmeister divided ions into kosmotropes and chaotropes based on their capacity to either “fit into” or “break” the ordered structure of water, and their capability to salt out or salt in proteins, respectively (Hofmeister, 1888). By arranging selected anions according to their impact on various protein properties, such as solubility, stability, or activity (Baldwin, 1996; Cacace et al., 1997; Dušeková et al., 2022; Sedlak et al., 2008), the so‐called Hofmeister series of anions can be arranged in the following order:

$${{\mathrm{SO}}_{4}}^{2-}>\begin{array}{l} {{\mathrm{HPO}}_{4}}^{2-}>F^{-}>{\mathrm{Cl}}^{-}>{\mathrm{Br}}^{-}>I^{-}>{{\mathrm{NO}}_{3}}^{-}\\ >{{\mathrm{ClO}}_{4}}^{-}>{\mathrm{SCN}}^{-}. \end{array}$$

In this series, chloride is considered “neutral” in terms of the Hofmeister effect. On the left side of chloride, the strongly hydrated anions are referred to as, “kosmotropic anions” due to the historical belief in promoting the structure of water and stabilizing the native structure of proteins. On the right side of chloride, weakly hydrated “chaotropic anions” are grouped; these anions disrupt the water structure and denature proteins. Omta et al. (2003) have shown that the effect of anions on water structure is negligible outside the hydration shell of an ion. Accordingly, numerous findings suggest that ions can interact directly with the first hydration shell of macromolecules and proteins in correlation with their position in the Hofmeister series, referred to as the Hofmeister effect (Hofmaier et al., 2023; Hofmeister, 1888; Kang et al., 2020; Mittal et al., 2019; Zhang & Cremer, 2006). Hydration of proteins affected by ion‐specific and ion‐unspecific effects plays a critical role in protein self‐assembly kinetics (Camino et al., 2021; Mittal et al., 2019; Sharma et al., 2018).

In the present work, the effect of nine sodium anions, namely, SO_4_^2−^, HPO_4_^2−^ (Pi), F^−^, Cl^−^, Br^−^, NO_3_^−^, I^−^, SCN^−^, and ClO_4_^−^, on the in vitro assembly of eADF4(C16) is examined. We show that only kosmotropic anions (SO_4_^2−^, Pi, F^−^) trigger fibril formation. To determine the self‐assembly mechanism of the eADF4(C16), we utilized the platform AmyloFit (Meisl et al., 2016) with integrated law kinetics. Global analysis elucidates a critical role of the secondary pathway represented by secondary nucleation for self‐assembly in the presence of kosmotropic anions at a concentration of 150 mM. On the contrary, the addition of chaotropic anions suppresses the self‐assembly in the presence of kosmotropic anions.

The mechanism of eADF4(C16) self‐assembly into fibrillar structures in presence of kosmotropic anions can be harnessed for designing scaffolds at micro‐ and nanoscales. These scaffolds can be utilized in tissue engineering, and regenerative medicine, as well as for a range of technical applications.

## 2 MATERIALS AND METHODS

All chemicals were purchased from (Carl Roth, Germany). Ultrapure water from a Millipore system (Merck KGaA, Germany) was used in the experiments.

### 2.1 Protein solubilization

The protein, eADF4(C16), was expressed in *E. coli* and purified as reported by Huemmerich et al. (2004). The structure of this protein is based on the C‐module with the amino acid sequence: GSSAAAAAAAASGPGGYGPENQGPSGPGGYGPGGP, which repeats 16 times. The protein was dissolved in a 6 M guanidinium thiocyanate solution and dialyzed against 10 mM Tris/HCl, pH 8.0 at room temperature. The buffer was changed four times every 2.5 h and once overnight. The protein solution was centrifuged using the ultracentrifuge (Optima MAX‐XP, Beckman‐Coulter, USA) at 185,000 g at 4°C for 50 min, and the protein concentration was determined using the UV–VIS spectrophotometer (NanoDrop 1000, Thermo Fisher, USA).

### 2.2 Kinetic measurements

Protein samples were prepared with a final concentration between 10 and 40 μM. Assays were initiated by adding 150 mM salts, namely, NaPi, KPi, Na_2_SO_4_, K_2_SO_4_, NaF, NaCl, NaI, NaBr, NaNO_3_, NaClO_4_, NaSCN, or in a combination of 150 mM KPi with other 150 mM, 300 mM, or 500 mM salt. For turbidity measurements, absorption at 340 nm was recorded on the spectrophotometer (Varian Cary 50 UV–Vis Spectrophotometer, Germany) at 20°C and in the 96‐well plate reader (SpectraMax iD5 Molecular Devices, USA) at 30°C every 10 min.

### 2.3 Pre‐formed fibrils (seeds)

Protein eADF4(C16) (at a final concentration of 20 μM) was incubated in 150 mM NaPi, at room temperature for 48 h to complete the fibril formation. Assembled fibrils were then sonicated using an ultrasonic homogenizer (Sonopuls HD 3200, Germany, MS73 tip set, 10% amplitude) for 15 s with six cycles while keeping the samples on ice to produce seeds. The seeds were added at 0.5% (w/w, seed/soluble protein) to protein solutions in the presence of 150 mM Na_2_SO_4_ or 150 mM NaF, and the change in the solution turbidity over time was recorded.

### 2.4 Curve fitting with Amylofit

Kinetic datasets of protein eADF4(C16) (10–40 μM) in the presence of 150 mM kosmotropic salt (Na_2_SO_4_, NaF) with or without seeds were fitted using the online platform AmyloFit (www.amylofit.ch.cam.ac.uk) (Meisl et al., 2016). The entire analysis was performed according to the protocol of Meisl et al. (2016). A detailed description is presented in the Supporting Information.

### 2.5 Circular dichroism spectroscopy (CD)

The far‐UV CD spectra were recorded by a spectropolarimeter (JASCO J‐815, Japan) with a Peltier cuvette holder at 20°C. The protein samples were diluted with water to a final concentration of 4 μM. Scans were obtained by averaging three individual spectra recorded between 190 and 250 nm, with points taken every 0.1 nm. Spectra of the buffers were subtracted from the spectra of the protein samples.

### 2.6 Fluorescence measurements

Thioflavin T (ThT) and 1‐anilino‐8‐naphthalene sulfonate acid (ANS) were prepared by dissolving in Milli‐Q water, followed by filtration utilizing a 0.2 mm syringe filter, with their concentrations determined through absorbance readings at 412 nm for ThT and 388 nm for ANS via a UV–visible spectrophotometer (Cary 50 UV, Varian, Germany). The molar extinction coefficients employed were 36,000 M^−1^.cm^−1^ for ThT and 5,200 M^−1^.cm^−1^ for ANS (Groenning et al., 2007; Ziaunys et al., 2019). Utilizing a plate‐reader (SpectraMax iD5, Molecular Devices, USA), fluorescence intensity was recorded for 30 μM Thioflavin‐T (ex. at 485 nm, em. at 528 nm) alongside a 15 μM protein solution and 150 mM kosmotropic or chaotropic salt, at intervals of 10 min at 30°C. Individual ANS spectra were obtained at a protein concentration of 10 μM, 150 mM chaotropic or kosmotropic salt, and 1 mM ANS, with excitation at 355 nm. Fluorescence spectra were documented utilizing a Shimadzu RF‐5000 spectrofluorimeter.

### 2.7 Transmission electron microscopy (TEM)

The protein samples (10 μL) were deposited on Pioloform‐carbon‐coated 200‐mesh copper grids (Plano GmBH, Germany) and incubated for 1 min at room temperature. The grids were washed with 5 μL MilliQ water and negatively stained with 2% uranyl acetate for 1 min. Images were recorded using a Zeiss LEO EM922 Omega microscope (Zeiss Microscopy, Jena, Germany), which operated at 200 kV accelerating voltage. The images were recorded by a bottom‐mounted CCD camera system (Ultrascan 1000, Gatan, Muenchen, Germany) and processed using a digital imaging processing system (Digital Micrograph GMS 1.9, Gatan, Muenchen, Germany).

## 3 RESULTS

### 3.1 Self‐assembly of the protein eADF4(C16) with kosmotropic or chaotropic salts

**FIGURE 1:**
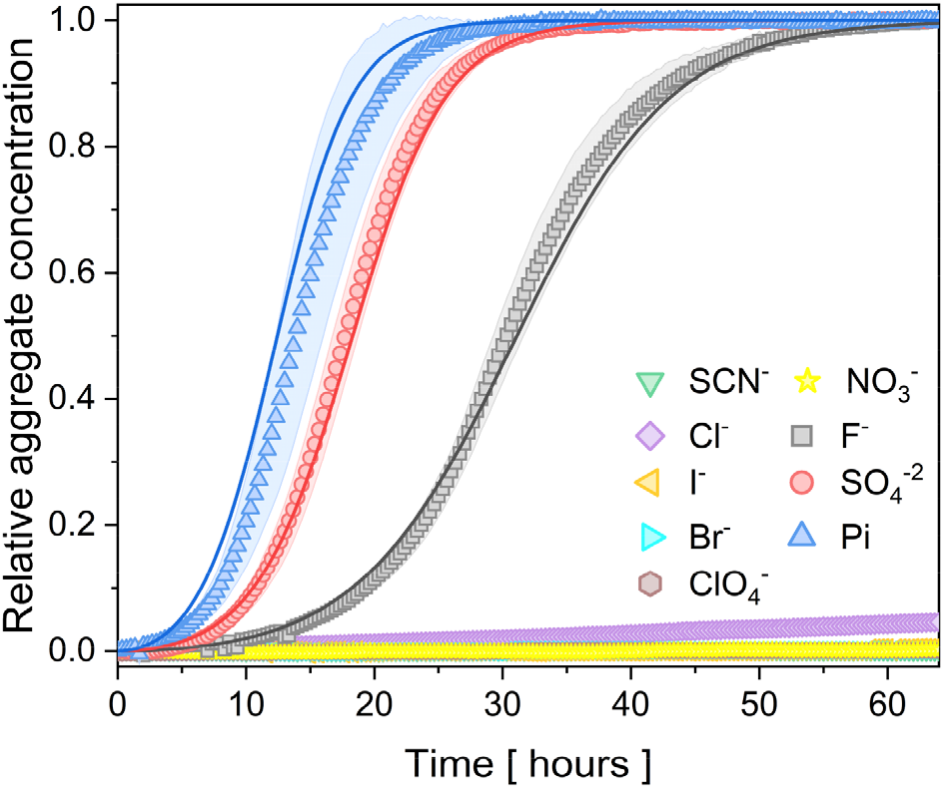
Aggregation kinetics of 20 μM eADF4(C16) protein in the presence of 150 mM kosmotropic or chaotropic salt at 20°C. Sigmoidal change of the turbidity at 340 nm corresponds to the self‐assembly of eADF4(C16) into nanofibrils. The presented data are an average of three replicates. Chaotropic salts (NaSCN, NaCl, NaI, NaBr, NaClO_4_, NaNO_3_) did not induce fibrillization. The kosmotropic salts were required to trigger nucleation, as well as fibril growth. The shaded area corresponds to standard deviation of the data. In some cases, the standard deviation is smaller than a symbol size. The curves were normalized between 0 and 1.

Self‐assembly of the protein eADF4(C16) in the presence of 150 mM kosmotropic or chaotropic salts was investigated by change of turbidity at 340 nm over at 20°C (Figure 1) as well as by detecting changes in Thioflavin‐T (ThT) fluorescence (Figure S1). ThT is a widely utilized fluorescent dye for monitoring the formation of cross‐beta sheet fibrils, owing to its substantial fluorescence enhancement upon attaching to the fibrils (Naiki et al., 1989; Sulatsky et al., 2020). Change of turbidity serves as another common method for studying protein fibrillization (Zhao et al., 2016). In this context, the similarity of the ThT and turbidity signals (Figure S1) validates the use of turbidity in the exploration of eADF4(C16) self‐assembly as it has been found out in a previous study by Humenik et al. (2014). It has been reported that a higher concentration of phosphate ions (>300 mM) led to the precipitation of the protein eADF4(C16) into particles, while low concentrations of Pi (<400 mM) trigger self‐assembly into nanofibrils (Humenik et al., 2015; Oktaviani et al., 2019; Slotta et al., 2008). Therefore, in the present study, a low concentration of phosphate salt (150 mM) was used to trigger the protein fibrillization. An analogous concentration effect on the protein fibril formation was observed for sulfate ions.

Sigmoidal turbidity evolution was recorded in the presence of kosmotropic ions (Pi, SO_4_^2−^, F^−^), indicating nucleated aggregation, independently of the cation (sodium or potassium) counterpart (Figure S2). This result is in good agreement with the observations of Humenik et al. (2015). Phosphate ions had the most pronounced effect on the process rate. No aggregation was detected upon the addition of chaotropic sodium anions (NO_3_^−^, Br^−^, I^−^, ClO_4_^−^, SCN^−^) or even the neutral Cl^−^ (from the Hofmeister effect point of view), indicating their stabilization effect on the soluble monomeric form of the eADF4(C16) protein (Figure 1).

### 3.2 Global analysis of eADF4(C16) aggregation at different concentrations in the presence of kosmotropic salts

The effect of kosmotropic ions (Pi, SO_4_^2−^, F^−^) on the self‐assembly of eADF4(C16) was studied in more detail. The change of turbidity at 340 nm was monitored across a range of protein concentrations (10–40 μM) in the presence of 150 mM phosphate, sulfate, or fluoride salts. Good reproducibility of experimental data measurements has been achieved by precisely defined experimental conditions (pH of the buffer, constant temperature of environment 20°C, and highly purified protein eADF4(C16) in monomeric form Hovanová et al., 2023). The kinetic measurements were performed in parallel using a single stock of protein solution.

**FIGURE 2:**
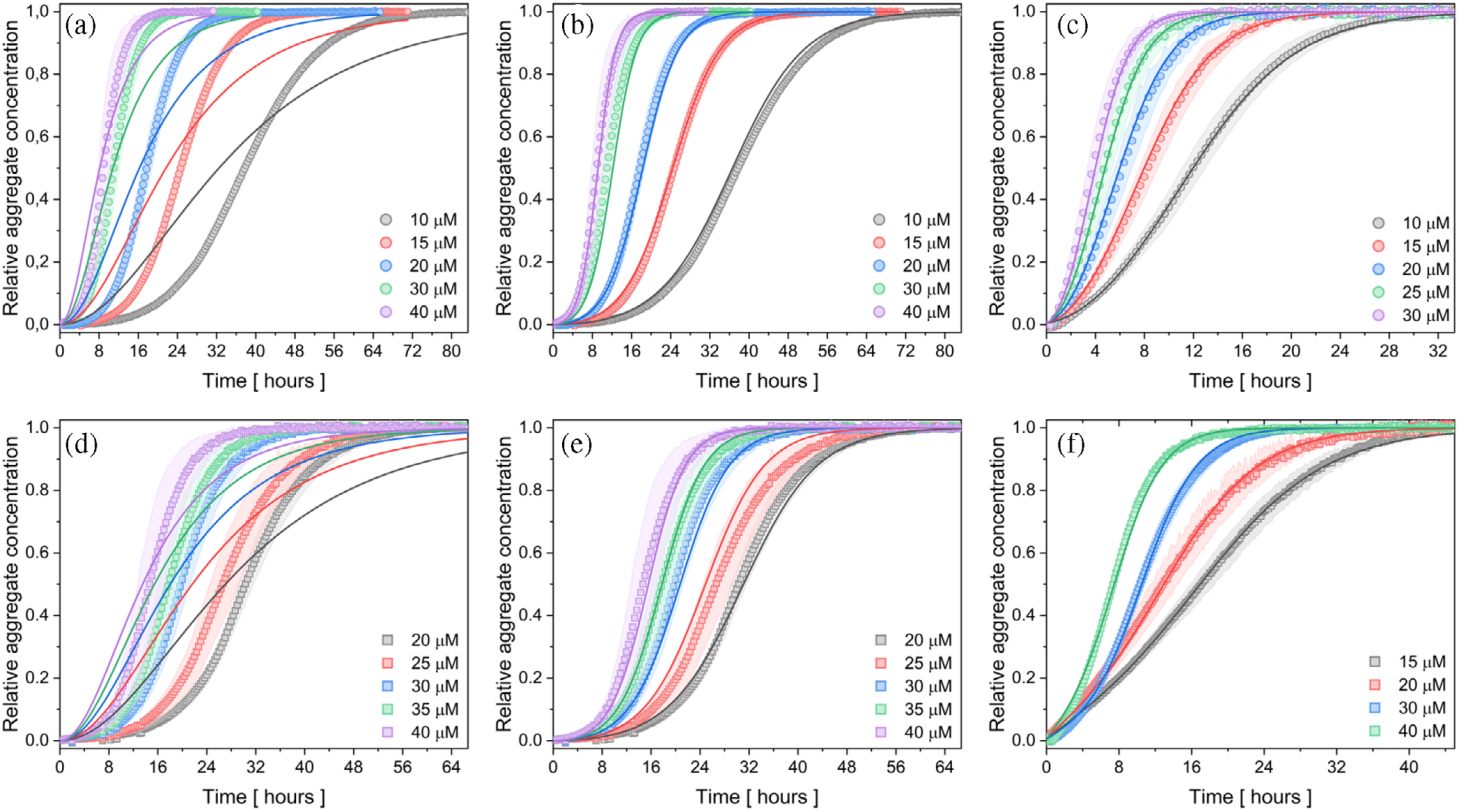
Kinetic measurements of 10–40 μM protein eADF4(C16) in 150 mM (a)–(c) Na_2_SO_4_, (d)–(f) NaF, without (a), (b), (d), (e) or with (c), (f) seeds at 20°C. Seeds concentration is 0.5% from the initial monomer protein concentration. For fitting of (a) and (d), a model of nucleation‐elongation was used, for (b), (c), (e), and (f), a model of secondary nucleation was employed. The normalized sigmoidal curves represent fibril formation, measured by turbidity at 340 nm. Note the different scales on the time axes. The presented data are the average of three replicates for each condition. The filled area corresponds to the standard deviation of the data, which in some cases is smaller than the symbol size.

A deeper understanding of the ion‐induced fibrillization of recombinant spider silk protein at the molecular level was provided by the analysis of kinetic data using the online platform Amylofit (Meisl et al., 2016), which enables the determination of the dominant mechanism of aggregation by global fitting. Based on the protocol (Meisl et al., 2016), the log–log plot of the half‐time (the data point corresponds to half of the signal between the initial line and the final plateau) against the monomer protein concentration for individual salts was created (Figure S3). The plots obtained for systems containing fluoride and sulfate ions were similar to those obtained for phosphate ions (Hovanová et al., 2023). Based on the corresponding power‐law dependence in log–log plots and negative scaling exponent, the simplest model of nucleation‐elongation (Figure 2a, d) was used to describe the self‐assembly of the protein eADF4(C16). Since the fitting result was unsatisfactory, a model that considers secondary pathways was used. The model of secondary nucleation fitted the kinetic curves with sufficient accuracy (Figure 2b, e), indicating its dominant role.

To verify the dominant secondary processes, a set of kinetic measurements was performed with the addition of nucleation seeds. The obtained data (Figure 2c, f) on 10–40 μM protein with the addition of 0.5% seeds in the presence of 150 mM NaF or 150 mM Na_2_SO_4_ were fitted with the same model of secondary nucleation as the set without seeds. The global fit of the given model accurately described the curves. The accuracy of the selected model was verified by the significant acceleration of fibrillization kinetics following the introduction of seeds, without capturing the initial lag phase. The dominance of the secondary nucleation mechanism was also shown by power function‐fits on a log–log graph with a linear slope (Figure S3c, d) and negative scaling exponents, with a −1 value in each case (Figure S3c, d). In the fitting procedure, the value of *n*_2_ was set to 1, according to the equation *γ* ≈ −(*n*_2_ + 1)/2 (Meisl et al., 2016), and *n*_*c*_ to 2, which is a recommended value in the Amylofit manual (https://amylofit.com/static/fitter_manual.pdf) and is also commonly used (Hovanová et al., 2023; Linse, 2021; Zhao et al., 2016). The model of secondary nucleation correctly described the course of measured kinetics with these parameter settings. Even when these parameters were set free, the fitting process resulted in converging to comparable values near 1 for *n*_2_ and 2 for *n*_*c*_, respectively.

The integral rate law (Cohen et al., 2011, 2012) enabled us to determine the values of the rate constants that control the reaction in the presence of fluoride and sulfate anions (Table 1). Although protein fibrillization involves rate constants for primary nucleation (*k*_*n*_), elongation (*k*_+_), and secondary nucleation (*k*_2_), the integrated rate law showed that the macroscopic reaction profiles for reactions beginning with monomeric protein are controlled by a combination of rate constants, *k*_+_ *k*_*n*_ and *k*_+_ *k*_2_. The global analyses shown in Figure 2b, e provided values for the combined rate parameters *k*_+_ *k*_*n*_ and *k*_+_ *k*_2_ (Table 1), which were shared globally. The absence of parameter errors is due to the convergence to the same value during the fitting procedure.

**TABLE 1:** Rate constants corresponding to the primary and secondary processes in the fibrillization of protein eADF4(C16) are shown in Figure 2.

Anion	*k*_+_ *k*_*n*_ [M^−2^ s^−2^]	*k*_+_ *k*_2_ [M^−2^ s^−2^]	*k*_+_ [M^−1^ s^−1^]	*k*_n_ [M^−1^ s^−1^]	*k*_2_ [M^−1^ s^−1^]
^a^Pi	5.00 × 10^6^	13.32 × 10^7^	12.48 × 10^7^	0.0401	1.578
SO_4_^2−^	1.35 × 10^6^	9.04 × 10^7^	2.85 × 10^7^	1.416	7.08
F^−^	0.42 × 10^6^	3.34 × 10^7^	7.26 × 10^7^	0.00585	0.5982

^a^ Values of rate constants corresponding to the protein self‐assembly in the presence of 150 mM Pi were taken from our recent work (Hovanová et al., 2023).

To determine the individual rate constants of the entire process from the combined rate parameters *k*_+_ *k*_*n*_ and *k*_+_ *k*_2_, the self‐assembly experiments from solutions containing not only protein monomers but also pre‐formed seeds were necessary to perform. The individual parameters, namely (i) *k*_*n*_—rate constant for nucleation, (ii) *k*_+_—rate constant for elongation and, (iii) *k*_2_—rate constant for secondary nucleation, were obtained by fitting the seeded datasets (Figure 2c, f) using the AmyloFit (Meisl et al., 2016) and are shown in Table 1. From the obtained values, it is seen that while elongation is the dominant event for fibrillization with 150 mM Pi and F^−^, in the case of SO_4_^2−^, it is secondary nucleation.

### 3.3 Morphology of nanofibrils

**FIGURE 3:**
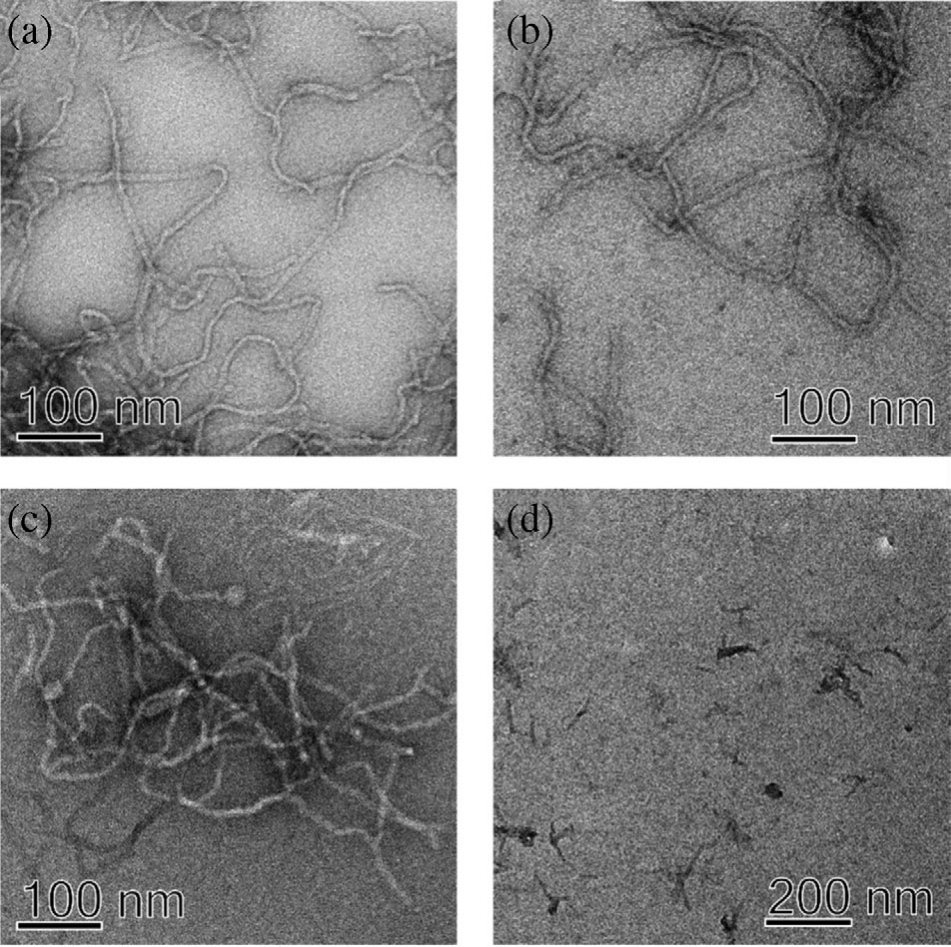
TEM images of fibrils formed after 48 h incubation in the presence of 150 mM (a) sodium phosphate, pH 8.0, (b) sodium sulfate, (c) sodium fluoride, at 20°C, without shaking. The image (d) shows short segments of fibrils, also called seeds, used in kinetic measurements for verification of the correct selection of the dominant mechanism (Figure 2c, f).

The structures formed in the presence of 150 mM Pi, F^−^, and SO_4_^2−^ were visualized using TEM after reaching the stationary phase (Figure 1). Clearly, regardless of the type of salt used, the resulting aggregates appear as fibrous, branched nanofibrils (Figure 3a–c). Figure 3d shows freshly prepared nucleation seeds that were used for verification of the chosen mechanism.

### 3.4 Competing effect of selected anions on Pi‐triggered eADF4(C16) fibrillization.

**FIGURE 4:**
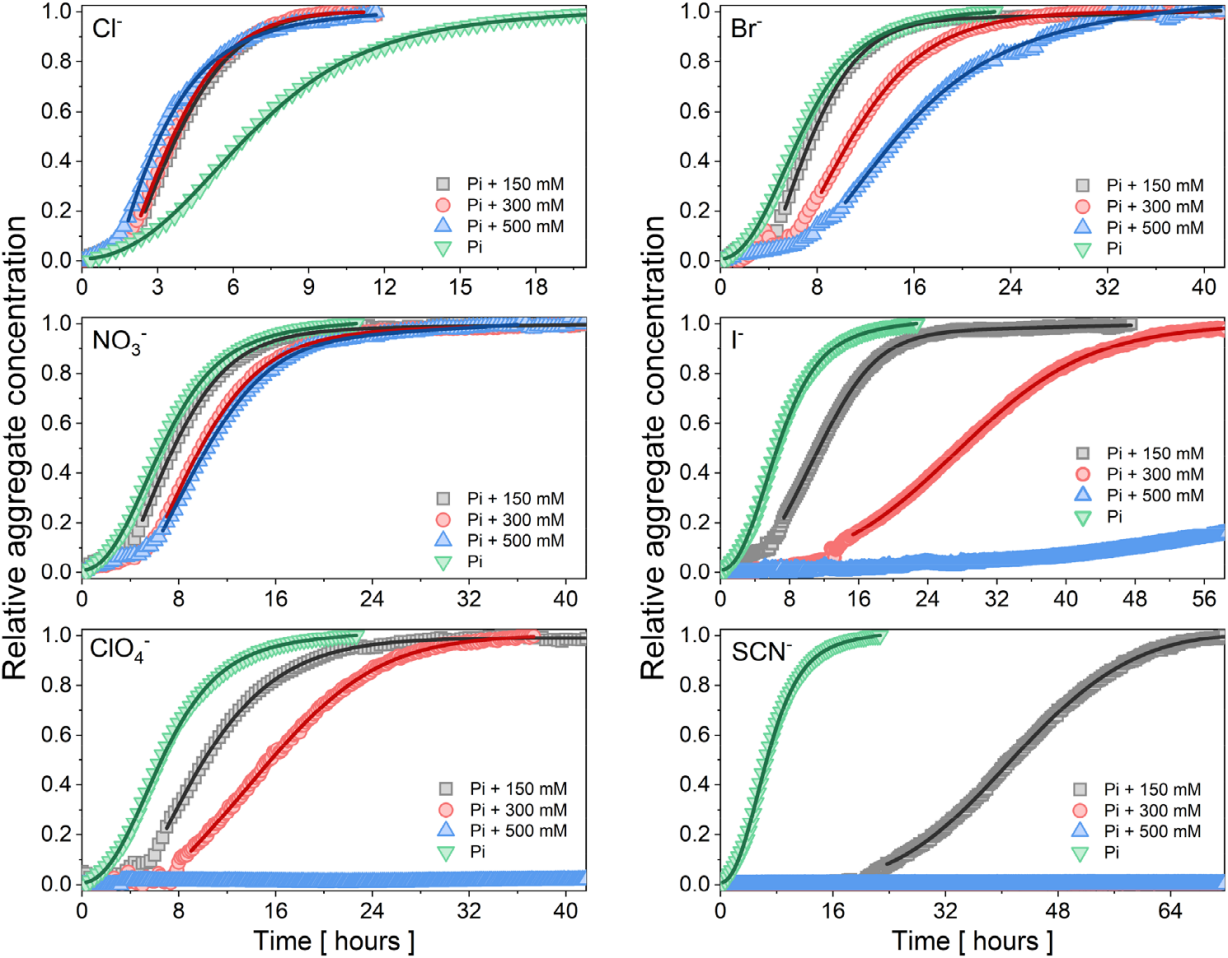
Influence of the chaotropic anions of the eADF4(C16) self‐assembly. The kinetic measurements at 15 μM protein and 150 mM KPi were performed in the presence of NaCl, NaNO_3_, NaBr, NaI, NaClO_4_, and NaSCN at 30°C. Every dataset comprises the condition with 150 mM KPi (green) and a combination of 150 mM KPi with 150 mM (gray), 300 mM (red), and 500 mM salt (blue). The normalized sigmoidal curves represent the turbidity evolution of the protein‐salt mixtures at 340 nm. Data were averaged over five replicates at each condition. Obtained dependencies were fitted by sigmoidal curves (Nielsen et al., 2001).

The effects of (Hofmeister) neutral (Cl^−^) and chaotropic anions (Br^−^, NO_3_^−^, I^−^, ClO_4_^−^, and SCN^−^) on the self‐assembly of eADF4(C16) in the presence of Pi, a commonly used trigger for protein fibril formation (Huemmerich et al., 2004; Slotta et al., 2008), were studied. As shown in Figure 4, only NaCl accelerated the self‐assembly process, while NaBr, NaNO_3_, NaI, NaClO_4_, and NaSCN suppressed it in a concentration‐dependent manner (150, 300, and 500 mM). Complete inhibition of protein self‐assembly occurred at 500 mM NaClO_4_ and 300 mM as well as 500 mM NaSCN. In these cases, the strong chaotropic anions eliminated the effect of kosmotropic phosphate anions. For comparison purposes, Figure S4 summarizes the effects of 500 mM studied salts in the mixture with 150 mM KPi on eADF4(C16) fibrillization: protein self‐assembly was fastest in the presence of Cl^−^, significantly slowed in the presence of Br^−^ > NO_3_^−^ > I^−^, and completely suppressed by SCN^−^ and ClO_4_^−^. These results are consistent with the position of anions in the Hofmeister series.

**FIGURE 5:**
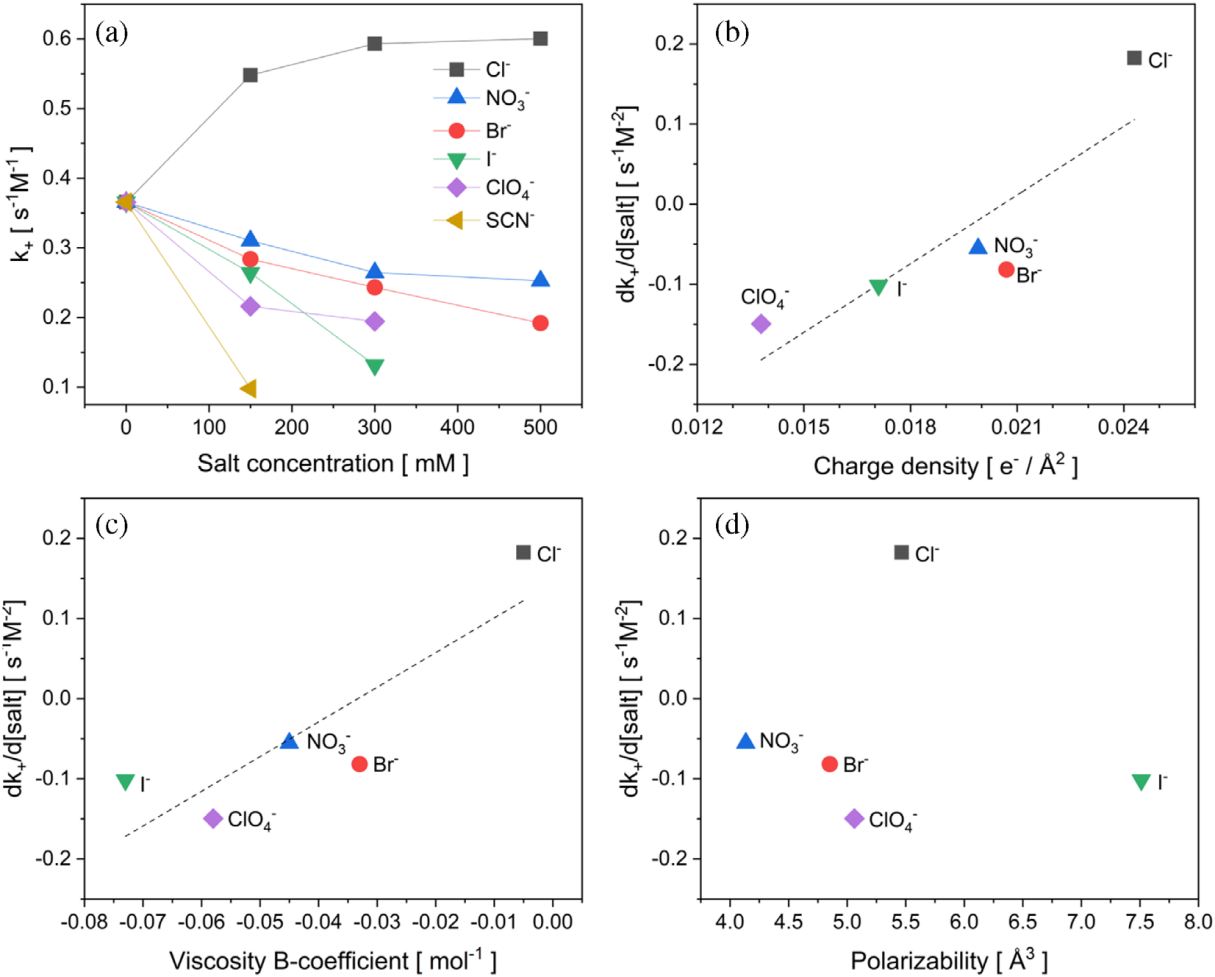
Correlations between the fibril elongation rate and anion properties. (a) Dependencies of the rate constant *k*_+_ (Table S1), as obtained by fitting the experiments in Figure 4a, on the salt concentrations. Parameters *dk*_+_/*d*[salt] obtained from (a) as a slope in the initial part (0–150 mM) were plotted in (b) versus charge density of the anions (dashed line represents linear correlation described by *y* = 28,597*x* − 0.589 and a correlation coefficient *R* ^2^ = 0.758); in (c) versus viscosity B‐coefficient (dashed linear shows linear correlation *y* = 4325*x* + 0.144 and a correlation coefficient *R* ^2^ = 0.743) and in (D) versus polarizability with no apparent correlation.

Anion effect on the aggregation kinetics was quantified in terms of elongation rate constants, as summarized in Table S1 and Figure 5. To determine whether there is a correlation between ion properties and their efficiency in affecting the rate of the eADF4(C16) fibril elongation, we plotted the slope of *dk*_+_/*d*[salt] from Figure 5a, within the concertation range of 0–150 mM salt, against selected intrinsic properties of the anions. Many parameters reflect different anion properties, but as we pointed out in our previous work, some of these parameters correlate with each other, significantly reducing the number of meaningful dependencies (Dušeková et al., 2022). As a result, we plotted the parameter *dk*_+_/*d*[salt] versus charge density (Figure 5b) (which also correlates with surface tension and air/water partition coefficient of anions), polarizability (Figure 5d), and viscosity B‐coefficient (which also correlates with partition coefficient at hydrocarbon surface) (Figure 5c). The dependencies indicate there are two relatively good correlations described by correlation coefficients *R* ^2^ > 0.7 of the parameter *dk*_+_/*d*[salt] versus the charge density and the viscosity B‐coefficient of anions.

### 3.5 Structural characterization of formed aggregates

The far‐UV CD spectra offer crucial insights into the proteins' secondary structure. The CD spectra of protein eADF4(C16) in the mixture of 150 mM KPi with sodium chloride (150, 300, 500 mM) and perchlorate (150, 300 mM) show the presence of β‐sheets by a pronounced negative ellipticity signal at ~220 nm and positive ellipticity at ~200 nm (Figure S5). The spectrum of the eADF4(C16) fibrils in 150 mM KPi solution was used for comparison, as the cross‐beta structure has been described previously under these conditions (Humenik et al., 2014, 2015; Slotta et al., 2008). The CD spectra strongly indicate: (i) the self‐assembly process of eADF4(C16) in the presence of KPi and chloride and perchlorate, representative of “neutral” and chaotropic anions, respectively, does not differ structurally and (ii) sodium perchlorate at 500 mM concentration stabilizes the unfolded water‐soluble conformation of the eADF4(C16), as indicated by the comparison of the spectrum to the freshly dialyzed protein. The effect of bromide, nitrate, iodide, and thiocyanate on the protein could not be analyzed due to high absorption in the far‐UV region.

To examine the presence of hydrophobic regions in the protein, eADF4(C16), incubated in the presence of kosmotropic or chaotropic salt, ANS‐induced fluorescence was employed since it binds to hydrophobic sites. ANS (8‐anilinonaphtalene‐1‐sulfonic acid) serves as a fluorescent probe for detecting hydrophobic regions on proteins (Sulatsky et al., 2020). The binding of ANS to hydrophobic patches on the protein's surface or to amorphous aggregates results in an emission shift from approximately 520 nm to about 465 nm, accompanied by a significant increase in fluorescence intensity. When the monomeric form of protein was introduced to ANS, only a minor increase in ANS fluorescence intensity was observed without a significant shift in the fluorescence peak (Figure S6). Similar signals were detected when the protein was incubated in chloride or perchloride anions. In contrast, the addition of eADF4(C16) fibrils led to a slight blue shift around 482 nm and a roughly three‐fold increase in ANS fluorescence intensity. These results infer that the monomeric form of eADF4(C16) lacks prominent binding sites for ANS, and there is only a modest increase in ANS binding to eADF4(C16) fibrils, in agreement with the conclusion of our very recent work (Hovanová et al., 2023).

## 4 DISCUSSION

The structural rearrangement of soluble, negatively charged, intrinsically disordered eADF4(C16) monomers into folded β‐sheet‐rich structures (Humenik et al., 2014) can occur through several different molecular processes in which ions can play a crucial role. Although Hofmeister‐ion effects on intrinsically disordered proteins are usually observed at higher salt concentrations (*≥*0.5 M), at lower concentrations (<0.5 M), nonspecific electrostatic screening is considered dominant (Gokarn et al., 2011; Maity et al., 2022). In case of eADF4(C16), the higher concentration of phosphate salt (>400 mM) typically lead to precipitation of protein into particles due to a decrease in the local flexibility of protein monomers caused by intermolecular interactions; low Pi concentrations (<300 mM) trigger self‐assembly into nanofibrils (Humenik et al., 2015; Oktaviani et al., 2019; Slotta et al., 2008). This study revealed that not only phosphate anions, but also other kosmotropic anions, such as sulfate and fluoride anions, promote self‐assembly with similar concentration limitations. A qualitative comparison showed previously that sulfate anions were slightly less efficient in triggering the eADF4(C16) fibril formation than phosphate anions (Humenik et al., 2015). Here, we confirmed this observation by quantification of the rate constants. Moreover, we showed for the first time that fluoride anions have surprisingly similar effects on protein self‐assembly as Pi and SO_4_^2−^, suggesting the size of the charge carried by anions is not the decisive feature of the anions' ability to trigger the fibrillization of eADF4(C16). In our very recent study on protein self‐assembly triggered by phosphate, we proved that, in addition to the typical primary pathways (primary nucleation and the fibril growth), a secondary nucleation pathway, where the monomers interact with the fibril surface played an important role in the assembly mechanism (Hovanová et al., 2023). The present investigation on the fibril formation in the presence of sulfate or fluoride ions confirmed the presence of the secondary pathway (Table 1). The accuracy of the selected model was backed by the successful global analysis of kinetic datasets of protein fibrillization with and without seeds. The absence of curvature in the log–log plots (Figure S3) further supported the involvement of only one dominant mechanism in the cross‐beta fibril formation. Observation of the fibrils using TEM showed that the resulting fibrils branched and differed slightly in diameter depending on the triggering ion. The obtained rate constants from the fitting of the kinetic datasets indicate that the proportion of individual events is different. During fibril formation with the addition of phosphate or fluoride, the primary elongation process dominated, although it was not possible to fit kinetic data without the secondary nucleation event. However, in the case of sulfate anions, the rate constant *k*_2_ increased by about one order of magnitude compared to fluoride and about five‐fold compared to phosphate. From these data, one can assume the resulting fibril morphology (branching density and the length of the branched fibrils) should differ. However, due to the highly entangled appearance of the fibril bundles in all three cases of the kosmotropic ion, statistically relevant evaluation of branching was impossible. On the other hand, attempts to disperse the fibrils from the cluster by ultrasonication or vigorous stirring induce energetic or shear forces, hence impacting fibril appearance. Nevertheless, sulfate ions can play a role in the preparation of fibril‐based hydrogels made of eADF4(C16) at higher protein concentrations (>1%) (Lechner et al., 2022; Schacht et al., 2015), resulting in branching into higher E‐moduli of the gels, impacting their processing via 3D printing or even cell differentiation in biofabrication procedures.

Although we determined a similar mechanism in all the studied cases of kosmotropic anions involved in eADF4(C16) self‐assembly, the question arises how these anions trigger the process of eADF4(C16) self‐assembly. The relatively low salt concentration (~150 mM) required to start the process suggests a direct protein‐anion interaction. On the other hand, the fact that the ions are structurally different and eADF4(C6) has polyanionic character given the negatively charged glutamic acid residue in the protein module, it weakens the argument for the direct protein–anion interaction. A recent detailed NMR study by Oktaviani et al. (2019) indicates the effect of kosmotropic anions on eADF4(C16) properties. In that work, the authors showed that kosmotropic anions increase the rigidity of the glycine region, promoting internal hydrogen bond interaction that leads to the formation of β‐sheet in the polyalanine region of the spider silk sequence. The findings of this study support the argument for an indirect anion interaction with protein through the Hofmeister effect. In the presence of kosmotropic anions, eADF4(C16) likely attains a specific self‐assembly‐prone conformation as a result of the preferential hydration of kosmotropic anions. In fact, for the formation of crystal‐like fibrils, the process requires proper protein conformation, in contrast to aggregation, which results in a protein amorphous structure (Ponikova et al., 2015; Zurdo et al., 2001). It needs to be stressed that the role of kosmotropic anions in this case principally differs from their role in the process of fibrillization or aggregation at acidic pH. In such cases, sulfate binds to the positively charged polypeptide chain and by counterbalancing strong repulsive electrostatic interactions, alleviates protein–protein interactions. Depending on the properties of the polypeptide chain and salt concentration, it can lead to the accelerated formation of amorphous aggregates and/or ordered fibrils (Buell et al., 2013; Klement et al., 2007; Owczarz & Arosio, 2014; Pedersen et al., 2006; Ruzafa et al., 2013). Another characteristic feature of anion‐induced fibrillization at acidic pH, which is different in the case of eADF4(C16) at neutral pH, is the efficiency of anion follows the electrosensitivity series, which orders the salts according to their retention times on anion exchange column (Gjerde et al., 1980), SO_4_^2−^ > ClO_4_^−^ > SCN^−^ > I^−^ > NO_3_^−^ > Br^−^ > Cl^−^ > H_2_PO_4_^−^ > F^−^, rather than the Hofmeister series, SO_4_^2−^ > H_2_PO_4_^−^ > F^−^ > Cl^−^ > Br^−^ > I^−^ > NO_3_^−^ > ClO_4_^−^ > SCN^−^.

Our results on the effect of salt mixtures suggest the chaotropic anions stabilize the eADF4(C16) water‐soluble form and prevent the formation of β‐sheet structure prone to self‐assembly. It has been demonstrated in numerous works that chaotropic anions likely bind to the peptide backbone of proteins and/or to the nonpolar group on the protein surface (Nandi & Robinson, 1972a, 1972b; Okur et al., 2017; Sedlak et al., 2008; Takekiyo et al., 2022). The direct binding of chaotropic anions to polypeptide chains is also supported by a correlation plot of the parameter *dk*_+_/*d*[salt] with intrinsic properties of anions, such as charge density and viscosity B‐coefficient (Figure 5), which indicates their partition at the water/protein interface and an important role of anion hydration for the protein‐anion interaction (Sedlak et al., 2008; Zhang & Cremer, 2010). This mechanism explains the inhibition and significant deceleration of fibril formation in the presence of chaotropic salts. As we showed, the efficiency of anions to decelerate the eADF4(C16) fibrillization correlates with their charge density, which likely corresponds to the affinity of anions to bind to the protein. Strong binding to the eADF4(C16) will stabilize the monomeric water‐soluble form of the protein and thus inhibits its fibrillization process. As such, we assume the deceleration of the eADF4(C16) fibrillation by salts and osmolytes can serve as an analytical tool for assessing the ability of these agents to bind to the protein structure as a result of their hydration properties. Following this line of reasoning, an intriguing exception was observed in the case of the chloride anions accelerating the self‐assembly of eADF4(C16) in the KPi buffer, unable to trigger but efficiently (positively) modulate the protein fibrillization. This observation is especially interesting in relation to the natural spinning process. In several studies, the role of chloride anions in the storage ampule of the spinning gland has been explained by their stabilizing effect on the soluble spidroins at high concentrations (50% w/v protein). Consequently, during the subsequent formation of the fiber in the channel, the exchange of chaotropic Na^+^ and Cl^−^ for kosmotropic K^+^ and Pi occurs (Arakawa et al., 2022; Knight & Vollrath, 2001; Oktaviani et al., 2019; Scheibel, 2004). However, the natural repetitive spidroin domains are flanked by folded, highly soluble C‐ and N‐terminal domains, which most probably significantly contribute to the stabilization of the whole protein in solution (Heidebrecht et al., 2015; Saric et al., 2021). Apparently, the neutral nature of chloride anions (from the Hofmeister effect point of view) did not influence the folding of the recombinant eADF4(C16) in the presence of Pi; increased Na^+^ concentration shielded the negative protein charge resulting in faster self‐assembly kinetic. As the natural spidroin involved globular terminal domain, as well as a much longer repetitive core comprising heterogeneous repetitive sequences, the self‐assembly of natural spider silk at physiological conditions, is a result of a complex interplay not only of the presence of kosmotropic/chaotropic ions but also other parameters, such as pH, water, and shear forces of the spinning process (Andersson et al., 2014; Knight & Vollrath, 2001; Scheibel, 2004).

## 5 CONCLUSION

**SCHEME 1:**
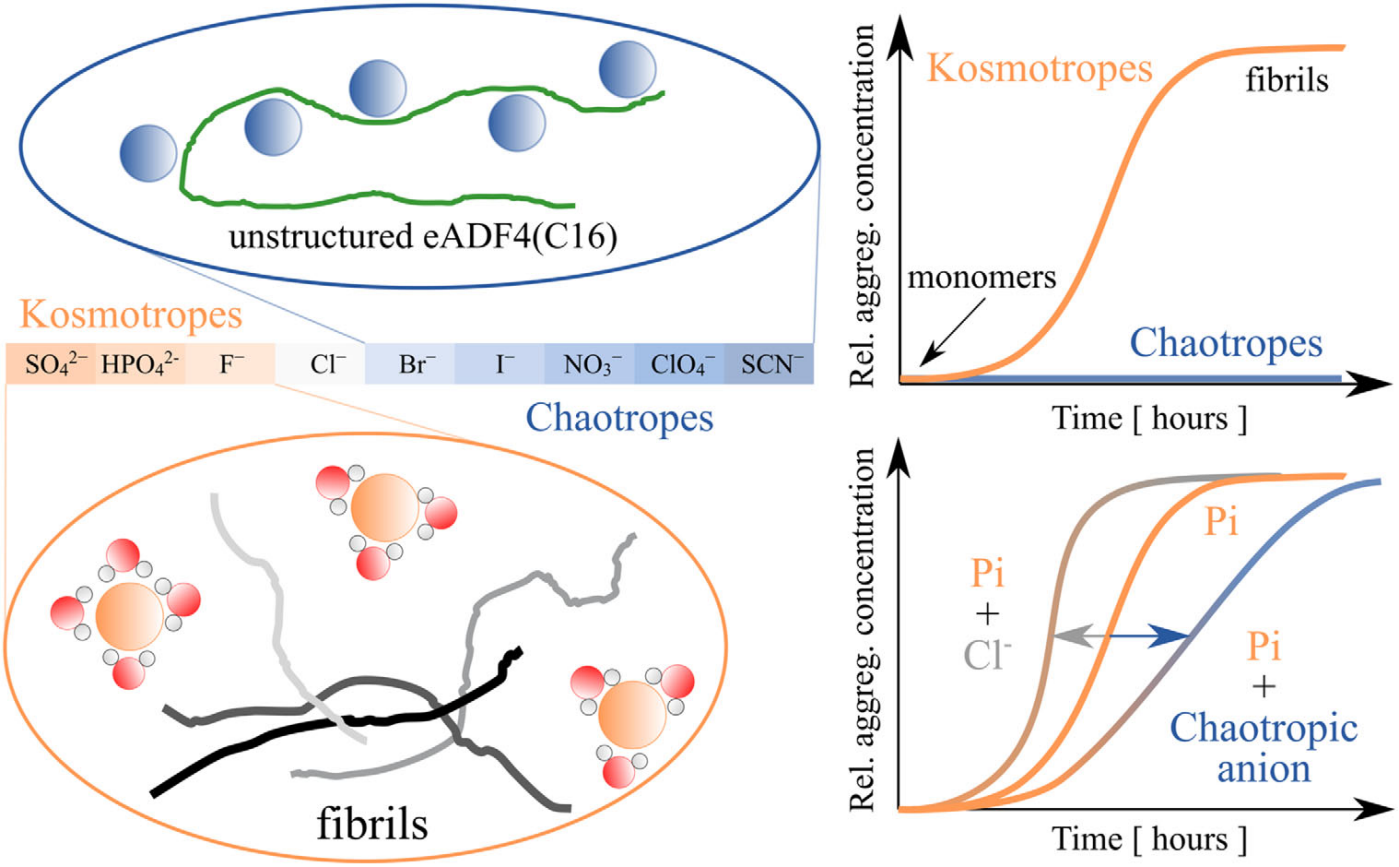
A schematic overview of the presented research illustrates the influence of kosmotropic and chaotropic anions, according to the Hofmeister order, on the self‐assembly of the protein, eADF4(C16). The water molecules form a structured arrangement around the kosmotropic anions, leading to the self‐assembly of the protein. The sigmoidal curves correspond to fibrillization process. In contrast, chaotropic anions stabilize the protein, possibly by binding to the protein structure as it remains dissolved in the solution. No increase in the intensity of the turbidity signal is observed during this event. The results of this study indicate that the addition of chloride anions to the kosmotropic phosphate salt accelerates the formation of fibrils. On the contrary, chaotropic anions slow down the fibrillization process or even inhibit it.

Our work provides a systematic in vitro study of the effect of Hofmeister anions on the fibrillization of the recombinant core spidroin eADF4(C16). We showed that similarly to phosphate anions, other kosmotropic anions such as sulfate and fluoride also trigger the fibrillization of the protein, and secondary nucleation plays an important role in the self‐assembly process. This result is likely achieved through the stabilization of fibrillization‐prone conformers of eADF4(C16) by anion hydration intermediates, in accordance with the Hofmeister effect. In contrast, chaotropic anions stabilize water‐soluble conformers that are unable to form fibrils, likely due to direct interaction with the polypeptide chain of eADF4(C16). Reported results further suggest that a hydrated cosolvent, for example, osmolytes, or crowding agents, may accelerate protein fibrillization (Scheme 1).

## AUTHOR CONTRIBUTIONS

**Erik Sedlák:** Conceptualization; formal analysis; writing – review and editing; funding acquisition. **Veronika Hovanová:** Investigation; formal analysis; writing – original draft. **Andrej Hovan:** Investigation; formal analysis; writing – original draft. **Martin Humenik:** Conceptualization; formal analysis; writing – review and editing; funding acquisition.

## CONFLICT OF INTEREST STATEMENT

The authors declare no conflicts of interest.

## Supporting information


**Data S1:** Supporting Information

## References

[pro4832-bib-0001] Andersson M , Chen GF , Otikovs M , Landreh M , Nordling K , Kronqvist N , et al. Carbonic anhydrase generates CO_2_ and H+ that drive spider silk formation via opposite effects on the terminal domains. PLoS Biol. 2014;12(8):e1001921. 10.1371/journal.pbio.1001921 25093327 PMC4122339

[pro4832-bib-0002] Arakawa K , Kono N , Malay AD , Tateishi A , Ifuku N , Masunaga H , et al. 1000 spider silkomes: linking sequences to silk physical properties. Science. 2022;8(41):eabo6043. 10.1126/sciadv.abo6043 PMC9555773 36223455

[pro4832-bib-0003] Baldwin RL . How Hofmeister ion interactions affect protein stability. Biophys J. 1996;71(4):2056–2063. 10.1016/s0006-3495(96)79404-3 8889180 PMC1233672

[pro4832-bib-0004] Buell AK , Hung P , Salvatella X , Welland ME , Dobson CM , Knowles TPJ . Electrostatic effects in filamentous protein aggregation. Biophys J. 2013;104(5):1116–1126. 10.1016/j.bpj.2013.01.031 23473495 PMC3610013

[pro4832-bib-0005] Cacace MG , Landau EM , Ramsden JJ . The Hofmeister series: salt and solvent effects on interfacial phenomena. Q Rev Biophys. 1997;30(3):241–277. 10.1017/s0033583597003363 9394422

[pro4832-bib-0006] Camino JD , Gracia P , Cremades N . The role of water in the primary nucleation of protein amyloid aggregation. Biophys Chem. 2021;269:106520. 10.1016/j.bpc.2020.106520 33341693

[pro4832-bib-0007] Cohen SIA , Vendruscolo M , Dobson CM , Knowles TPJ . Nucleated polymerization with secondary pathways. II. Determination of self‐consistent solutions to growth processes described by non‐linear master equations. J Chem Phys. 2011;135(6):065106. 10.1063/1.3608917 21842955 PMC5036541

[pro4832-bib-0008] Cohen SIA , Vendruscolo M , Dobson CM , Knowles TPJ . From macroscopic measurements to microscopic mechanisms of protein aggregation [review]. J Mol Biol. 2012;421(2–3):160–171. 10.1016/j.jmb.2012.02.031 22406275

[pro4832-bib-0009] Collins KD . Ions from the Hofmeister series and osmolytes: effects on proteins in solution and in the crystallization process. Methods. 2004;34(3):300–311. 10.1016/j.ymeth.2004.03.021 15325648

[pro4832-bib-0010] de Oliveira DH , Biler M , Mim C , Enstedt L , Kvick M , Norman P , et al. Silk assembly against hydrophobic surfaces‐modeling and imaging of formation of nanofibrils. ACS Appl Bio Mater. 2023;6(3):1011–1018. 10.1021/acsabm.2c00878 PMC10031558 36791416

[pro4832-bib-0011] Dušeková E , Garajová K , Yavaşer R , Tomková M , Sedláková D , Dzurillová V , et al. Modulation of global stability, ligand binding and catalytic properties of trypsin by anions. Biophys Chem. 2022;288:106856. 10.1016/j.bpc.2022.106856 35872468

[pro4832-bib-0012] Gaspar VM , Lavrador P , Borges J , Oliveira MB , Mano JF . Advanced bottom‐up engineering of living architectures. Advanced Materials. 2020;32(6):1903975. 10.1002/adma.201903975 31823448

[pro4832-bib-0013] Gjerde DT , Schmuckler G , Fritz JS . Anion chromatography with low‐conductivity eluents. II. J Chromatogr. 1980;187(1):35–45. 10.1016/s0021-9673(00)87871-1

[pro4832-bib-0014] Gokarn YR , Fesinmeyer RM , Saluja A , Razinkov V , Chase SF , Laue TM , et al. Effective charge measurements reveal selective and preferential accumulation of anions, but not cations, at the protein surface in dilute salt solutions. Protein Sci. 2011;20(3):580–587. 10.1002/pro.591 21432935 PMC3064836

[pro4832-bib-0015] Groenning M , Norrman M , Flink JM , van de Weert M , Bukrinsky JT , Schluckebier G , et al. Binding mode of thioflavin T in insulin amyloid fibrils. J Struct Biol. 2007;159(3):483–497. 10.1016/j.jsb.2007.06.004 17681791

[pro4832-bib-0016] Heidebrecht A , Eisoldt L , Diehl J , Schmidt A , Geffers M , Lang G , et al. Biomimetic fibers made of recombinant spidroins with the same toughness as natural spider silk. Adv Mater. 2015;27(13):2189–2194. 10.1002/adma.201404234 25689835

[pro4832-bib-0017] Hofmaier M , Malanin M , Bittrich E , Lentz S , Urban B , Scheibel T , et al. β‐sheet structure formation within binary blends of two spider silk related p eptides. Biomacromolecules. 2023;24(2):825–840. 10.1021/acs.biomac.2c01266 36632028

[pro4832-bib-0018] Hofmeister F . Zur Lehre von der Wirkung der Salze. Archiv für Experimentelle Pathologie Und Pharmakologie. 1888;24(4):247–260. 10.1007/BF01918191

[pro4832-bib-0019] Hovanová V , Hovan A , Žoldák G , Sedlák E , Humenik M . Global analysis of kinetics reveals the role of secondary nucleation in recombinant spider silk self‐assembly. Protein Sci. 2023;8:e4722. 10.1002/pro.4722 PMC10364585 37417849

[pro4832-bib-0020] Huemmerich D , Helsen CW , Quedzuweit S , Oschmann J , Rudolph R , Scheibel T . Primary structure elements of spider dragline silks and their contribution to protein solubility. Biochemistry. 2004;43(42):13604–13612. 10.1021/bi048983q 15491167

[pro4832-bib-0021] Humenik M , Magdeburg M , Scheibel T . Influence of repeat numbers on self‐assembly rates of repetitive recombinant spider silk proteins. J Struct Biol. 2014;186(3):431–437. 10.1016/j.jsb.2014.03.010 24657229

[pro4832-bib-0022] Humenik M , Smith AM , Arndt S , Scheibel T . Ion and seed dependent fibril assembly of a spidroin core domain [article]. J Struct Biol. 2015;191(2):130–138. 10.1016/j.jsb.2015.06.021 26123261

[pro4832-bib-0023] Humenik M , Smith AM , Scheibel T . Recombinant spider silks‐biopolymers with potential for future applications. Polymers. 2011;3(1):640–661. 10.3390/polym3010640

[pro4832-bib-0024] Humenik M , Winkler A , Scheibel T . Patterning of protein‐based materials. Biopolymers. 2021;112(2):e23412. 10.1002/bip.23412 33283876

[pro4832-bib-0025] Kamada A , Toprakcioglu Z , Knowles TPJ . Kinetic analysis reveals the role of secondary nucleation in regenerated silk fibroin self‐assembly. Biomacromolecules. 2023;24(4):1709–1716. 10.1021/acs.biomac.2c01479 36926854 PMC10091410

[pro4832-bib-0026] Kang BB , Tang HC , Zhao ZD , Song SS . Hofmeister series: insights of ion specificity from amphiphilic assembly and interface property. ACS Omega. 2020;5(12):6229–6239. 10.1021/acsomega.0c00237 32258857 PMC7114165

[pro4832-bib-0027] Kim SH , Hong H , Ajiteru O , Sultan MT , Lee YJ , Lee JS , et al. 3D bioprinted silk fibroin hydrogels for tissue engineering. Nat Protoc. 2021;16(12):5484–5532. 10.1038/s41596-021-00622-1 34716451

[pro4832-bib-0028] Klement K , Wieligmann K , Meinhardt J , Hortschansky P , Richter W , Fandrich M . Effect of different salt ions on the propensity of aggregation and on the structure of Alzheimer's A beta(1‐40) amyloid fibrils. J Mol Biol. 2007;373(5):1321–1333. 10.1016/j.jmb.2007.08.068 17905305

[pro4832-bib-0029] Knight DP , Vollrath F . Changes in element composition along the spinning duct in a Nephila spider. Naturwissenschaften. 2001;88(4):179–182. 10.1007/s001140100220 11480706

[pro4832-bib-0030] Lamberger Z , Kocourkova K , Minarik A , Humenik M . Dual patterning of self‐assembling spider silk protein nanofibrillar networks. Adv Mater Interfaces. 2022;9(31):2201173. 10.1002/admi.202201173

[pro4832-bib-0031] Leal‐Egana A , Scheibel T . Silk‐based materials for biomedical applications. Biotechnol Appl Biochem. 2010;55:155–167. 10.1042/ba20090229 20222871

[pro4832-bib-0032] Lechner A , Trossmann VT , Scheibel T . Impact of cell loading of recombinant spider silk based bioinks on gelation and printability. Macromol Biosci. 2022;22(3):2100390. 10.1002/mabi.202100390 34882980

[pro4832-bib-0033] Lendel C , Solin N . Protein nanofibrils and their use as building blocks of sustainable materials. RSC Adv. 2021;11(62):39188–39215. 10.1039/d1ra06878d 35492452 PMC9044473

[pro4832-bib-0034] Ling SJ , Kaplan DL , Buehler MJ . Nanofibrils in nature and materials engineering. Nat Rev Mater. 2018;3(4):18016. 10.1038/natrevmats.2018.16 34168896 PMC8221570

[pro4832-bib-0035] Linse S . Toward the equilibrium and kinetics of amyloid peptide self‐assembly. Curr Opin Struct Biol. 2021;70:87–98. 10.1016/j.sbi.2021.05.004 34153659

[pro4832-bib-0036] Lombardo D , Calandra P , Pasqua L , Magazu S . Self‐assembly of organic nanomaterials and biomaterials: the bottom‐up approach for functional nanostructures formation and advanced applications. Materials. 2020;13(5):1048. 10.3390/ma13051048 32110877 PMC7084717

[pro4832-bib-0037] Maity H , Baidya L , Reddy G . Salt‐induced transitions in the conformational ensembles of intrinsically disordered proteins. J Phys Chem B. 2022;126(32):5959–5971. 10.1021/acs.jpcb.2c03476 35944496

[pro4832-bib-0038] Meisl G , Kirkegaard JB , Arosio P , Michaels TCT , Vendruscolo M , Dobson CM , et al. Molecular mechanisms of protein aggregation from global fitting of kinetic models. Nat Protoc. 2016;11(2):252–272. 10.1038/nprot.2016.010 26741409

[pro4832-bib-0039] Mittal N , Benselfelt T , Ansari F , Gordeyeva K , Roth SV , Wagberg L , et al. Ion‐specific assembly of strong, tough, and stiff biofibers. Angew Chem‐Int Ed. 2019;58(51):18562–18569. 10.1002/anie.201910603 PMC6916401 31600016

[pro4832-bib-0040] Muller‐Herrmann S , Scheibel T . Enzymatic degradation of films, particles, and nonwoven meshes made of a recombinant spider silk protein. ACS Biomater Sci Eng. 2015;1(4):247–259. 10.1021/ab500147u 33435049

[pro4832-bib-0041] Naiki H , Higuchi K , Hosokawa M , Takeda T . Fluorometric‐determination of amyloid fibrils invitro using the fluorescent dye thioflavine‐T. Anal Biochem. 1989;177(2):244–249. 10.1016/0003-2697(89)90046-8 2729542

[pro4832-bib-0042] Nandi PK , Robinson DR . Effects of salts on free‐energies of nonpolar groups in model peptides. J Am Chem Soc. 1972a;94(4):1308–1315. 10.1021/ja00759a043 5060274

[pro4832-bib-0043] Nandi PK , Robinson DR . Effects of salts on free‐energy of peptide group. J Am Chem Soc. 1972b;94(4):1299–1308. 10.1021/ja00759a042 5060273

[pro4832-bib-0044] Nielsen L , Khurana R , Coats A , Frokjaer S , Brange J , Vyas S , et al. Effect of environmental factors on the kinetics of insulin fibril formation: Elucidation of the molecular mechanism. Biochemistry. 2001;40(20):6036–6046. 10.1021/bi002555c 11352739

[pro4832-bib-0045] Oktaviani NA , Matsugami A , Hayashi F , Numata K . Ion effects on the conformation and dynamics of repetitive domains of a spider silk protein: implications for solubility and beta‐sheet formation. Chem Commun. 2019;55(66):9761–9764. 10.1039/c9cc03538a 31355386

[pro4832-bib-0046] Okur HI , Hladílková J , Rembert KB , Cho Y , Heyda J , Dzubiella J , et al. Beyond the Hofmeister series: ion‐specific effects on proteins and their biological functions. J Phys Chem B. 2017;121(9):1997–2014. 10.1021/acs.jpcb.6b10797 28094985

[pro4832-bib-0047] Olson E , Liu F , Blisko J , Li YF , Tsyrenova A , Mort R , et al. Self‐assembly in biobased nanocomposites for multifunctionality and improved performance. Nanoscale Adv. 2021;3(15):4321–4348. 10.1039/d1na00391g 36133470 PMC9418702

[pro4832-bib-0048] Omta AW , Kropman MF , Woutersen S , Bakker HJ . Negligible effect of ions on the hydrogen‐bond structure in liquid water. Science. 2003;301(5631):347–349. 10.1126/science.1084801 12869755

[pro4832-bib-0049] Owczarz M , Arosio P . Sulfate anion delays the self‐assembly of human insulin by modifying the aggregation pathway. Biophys J. 2014;107(1):197–207. 10.1016/j.bpj.2014.05.030 24988354 PMC4119273

[pro4832-bib-0050] Pedersen JS , Flink JM , Dikov D , Otzen DE . Sulfates dramatically stabilize a salt‐dependent type of glucagon fibrils. Biophys J. 2006;90(11):4181–4194. 10.1529/biophysj.105.070912 16533857 PMC1459509

[pro4832-bib-0051] Percebom AM , Towesend VJ , Pereira M , Gramatges AP . Sustainable self‐assembly strategies for emerging nanomaterials. Curr Opin Green Sustain Chem. 2018;12:8–14. 10.1016/j.cogsc.2018.04.004

[pro4832-bib-0052] Poniková S , Antošová A , Demjén E , Sedláková D , Marek J , Varhač R , et al. Lysozyme stability and amyloid fibrillization dependence on Hofmeister anions in acidic pH. JBIC. 2015;20(6):921–933. 10.1007/s00775-015-1276-0 26077813

[pro4832-bib-0053] Rahamtullah , Mishra R . Nicking and fragmentation are responsible for alpha‐lactalbumin amyloid fibril formation at acidic pH and elevated temperature. Protein Sci. 2021;30(9):1919–1934. 10.1002/pro.4144 34107116 PMC8376409

[pro4832-bib-0054] Raman B , Chatani E , Kihara M , Ban T , Sakai M , Hasegawa K , et al. Critical balance of electrostatic and hydrophobic interactions is required for beta(2)‐microglobulin amyloid fibril growth and stability. Biochemistry. 2005;44(4):1288–1299. 10.1021/bi048029t 15667222

[pro4832-bib-0055] Rammensee S , Slotta U , Scheibel T , Bausch AR . Assembly mechanism of recombinant spider silk proteins. Proc Natl Acad Sci USA. 2008;105(18):6590–6595. 10.1073/pnas.0709246105 18445655 PMC2373321

[pro4832-bib-0056] Ruzafa D , Conejero‐Lara F , Morel B . Modulation of the stability of amyloidogenic precursors by anion binding strongly influences the rate of amyloid nucleation. Phys Chem Chem Phys. 2013;15(37):15508–15517. 10.1039/c3cp52313f 23942905

[pro4832-bib-0057] Saric M , Eisoldt L , Doring V , Scheibel T . Interplay of different major Ampullate Spidroins during assembly and implications for fiber mechanics. Adv Mater. 2021;33(9):2006499. 10.1002/adma.202006499 PMC11468934 33496360

[pro4832-bib-0058] Schacht K , Jungst T , Schweinlin M , Ewald A , Groll J , Scheibel T . Biofabrication of cell‐loaded 3D spider silk constructs. Angew Chem‐Int Ed. 2015;54(9):2816–2820. 10.1002/anie.201409846 25640578

[pro4832-bib-0059] Scheibel T . Spider silks: recombinant synthesis, assembly, spinning, and engineering of synthetic proteins. Microb Cell Fact. 2004;3:14. 10.1186/1475-2859-3-14 15546497 PMC534800

[pro4832-bib-0060] Scheibel T , Trossmann VT , Lechner A , Bargel H , Humenik M , Žurovec M . Bioinspirierte Klebstoffe zur Anwendung in wässrigen Flüssigkeiten. Adhaes Kleb Dicht. 2022;66(1):34–39. 10.1007/s35145-022-0554-6

[pro4832-bib-0061] Sedlak E , Stagg L , Wittung‐Stafshede P . Effect of Hofmeister ions on protein thermal stability: roles of ion hydration and peptide groups? Arch Biochem Biophys. 2008;479(1):69–73. 10.1016/j.abb.2008.08.013 18782555

[pro4832-bib-0062] Sharma A , Behrens SH , Chernoff YO , Bommarius AS . Modulation of the formation of a beta‐ and Sup35NM‐based amyloids by complex interplay of specific and nonspecific ion effects. J Phys Chem B. 2018;122(19):4972–4981. 10.1021/acs.jpcb.7b12836 29668283 PMC6932987

[pro4832-bib-0063] Shen Y , Levin A , Kamada A , Toprakcioglu Z , Rodriguez‐Garcia M , Xu YF , et al. From protein building blocks to functional materials. ACS Nano. 2021;15(4):5819–5837. 10.1021/acsnano.0c08510 33760579 PMC8155333

[pro4832-bib-0064] Slotta U , Hess S , Spiess K , Stromer T , Serpell L , Scheibel T . Spider silk and amyloid fibrils: a structural comparison. Macromol Biosci. 2007;7(2):183–188. 10.1002/mabi.200600201 17295405

[pro4832-bib-0065] Slotta UK , Rammensee S , Gorb S , Scheibel T . An engineered spider silk protein forms microspheres. Angew Chem‐Int Ed. 2008;47(24):4592–4594. 10.1002/anie.200800683 18461576

[pro4832-bib-0066] Steiner D , Lang G , Fischer L , Winkler S , Fey T , Greil P , et al. Intrinsic vascularization of recombinant eADF4(C16) spider silk matrices in the arteriovenous loop model. Tissue Eng Part A. 2019;25(21–22):1504–1513. 10.1089/ten.tea.2018.0360 30848159

[pro4832-bib-0067] Steiner D , Winkler S , Heltmann‐Meyer S , Trossmann VT , Fey T , Scheibel T , et al. Enhanced vascularization and de novo tissue formation in hydrogels made of engineered RGD‐tagged spider silk proteins in the arteriovenous loop model. Biofabrication. 2021;13(4):045003. 10.1088/1758-5090/ac0d9b 34157687

[pro4832-bib-0068] Su Y , Chang PT . Acidic pH promotes the formation of toxic fibrils from beta‐amyloid peptide. Brain Res. 2001;893(1–2):287–291. 10.1016/s0006-8993(00)03322-9 11223020

[pro4832-bib-0069] Sulatsky MI , Sulatskaya AI , Povarova OI , Antifeeva IA , Kuznetsova IM , Turoverov KK . Effect of the fluorescent probes ThT and ANS on the mature amyloid fibrils. Prion. 2020;14(1):67–75. 10.1080/19336896.2020.1720487 32008441 PMC7009331

[pro4832-bib-0070] Sun H , Marelli B . Polypeptide templating for designer hierarchical materials. Nat Commun. 2020;11(1):351. 10.1038/s41467-019-14257-0 31953407 PMC6969164

[pro4832-bib-0071] Takekiyo T , Yamada N , Amo T , Asano A , Yoshimura Y . Tuning magnetic heating efficiency of colloidal dispersions of iron oxide nano‐clusters by varying the surfactant concentration during solvothermal synthesis. J Mol Liq. 2022;360:119446. 10.1016/j.molliq.2022.119446

[pro4832-bib-0072] Trossmann VT , Scheibel T . Design of recombinant spider silk proteins for cell type specific binding. Adv Healthc Mater. 2023;12(9):2202660. 10.1002/adhm.202202660 PMC11468868 36565209

[pro4832-bib-0073] Wegst UGK , Bai H , Saiz E , Tomsia AP , Ritchie RO . Bioinspired structural materials. Nat Mater. 2015;14(1):23–36. 10.1038/nmat4089 25344782

[pro4832-bib-0074] Weisenberger MS , Deans TL . Bottom‐up approaches in synthetic biology and biomaterials for tissue engineering applications. J Ind Microbiol Biotechnol. 2018;45(7):599–614. 10.1007/s10295-018-2027-3 29552703 PMC6041164

[pro4832-bib-0075] Zhang YJ , Cremer PS . Interactions between macromolecules and ions: the Hofmeister series. Curr Opin Chem Biol. 2006;10(6):658–663. 10.1016/j.cbpa.2006.09.020 17035073

[pro4832-bib-0076] Zhang YJ , Cremer PS . Chemistry of Hofmeister anions and osmolytes. Annu Rev Phys Chem. 2010;61:63–83. 10.1146/annurev.physchem.59.032607.093635 20055667

[pro4832-bib-0077] Zhao R , So M , Maat H , Ray NJ , Arisaka F , Goto Y , et al. Measurement of amyloid formation by turbidity assay—seeing through the cloud. Biophys Rev. 2016;8(4):445–471. 10.1007/s12551-016-0233-7 28003859 PMC5135725

[pro4832-bib-0078] Ziaunys M , Mikalauskaite K , Smirnovas V . Amyloidophilic molecule interactions on the surface of insulin fibrils: cooperative binding and fluorescence quenching. Sci Rep. 2019;9:20303. 10.1038/s41598-019-56788-y 31889118 PMC6937241

[pro4832-bib-0079] Zurdo J , Guijarro JI , Jimenez JL , Saibil HR , Dobson CM . Dependence on solution conditions of aggregation and amyloid formation by an SH3 domain. J Mol Biol. 2001;311(2):325–340. 10.1006/jmbi.2001.4858 11478864

