# Supplementary material for: Only kosmotrope anions trigger fibrillization of the recombinant core spidroin eADF4(C16) from Araneus diadematus
Source: Protein Sci. 2023 Dec 1;32(12):e4832. doi: 10.1002/pro.4832 (PMC10661072; doi:10.1002/pro.4832)
Supplement: Supplementary file 1 — Data S1: Supporting Information [file PRO-32-e4832-s001.docx]

Supporting information

to

**Only kosmotrope anions trigger fibrillization of the recombinant core spidroin eADF4(C16) from *Araneus diadematus***

Veronika Hovanová, Andrej Hovan, Martin Humenik, Erik Sedlák

Supporting Procedures

*Curve fitting with Amylofit.* The normalized kinetic data were uploaded to the platform and the half-times of eADF4(C16) self-assembly were generated. The half-time values correspond to the time point of each curve, at which the intensity of turbidity reaches half of the maximum signal between the baseline and the plateau. Based on the double logarithmic graph of the dependence of half-times ($\tau$) on the initial protein monomer concentrations (*m*_0_) the scaling exponent (γ), which shows the slope of the double logarithmic plot, was determined. Both variables are reproduced by $\log(\tau)=\gamma\log\left( m_{0} \right)+constant$. Linear slope (power function dependence) indicates the independence of scaling exponent on monomer concentration. Deviations of the points from a straight line mean that the dominant mechanism of aggregation is changing with monomer concentration. The concave plot represents negative curvature, indicating parallel competitive processes. The convex plot has positive curvature, suggesting the presence of a saturation effect. Based on the scaling exponent, possible models are selected. In any case, AmyloFit considers models of molecular mechanism involving nucleation and growth processes. The platform describes two quantities: the aggregate number concentration, *P*(*t*), and the aggregate mass concentration, *M*(*t*). All microscopic processes can be divided into two groups, those that change the aggregate number or mass.

Kinetic models in AmyloFit consider three processes that affect the aggregate number concentration: (i) Primary nucleation, representing homogeneous nucleation in solution, is described by a reaction order (*n*_c_) and rate constant (*k*_n_); (ii) Secondary nucleation is defined by the reaction order (*n*_2_) as well as the rate constants (*k*_2_), and an example is surface-catalyzed nucleation, i.e., new aggregates are nucleated on the surface of existing fibrils; (iii) Fragmentation depends only on the concentration of aggregate mass, has reaction order 1 and rate constant (*k*_-_). The rate constant for elongation (*k_+_*) is considered in all cases.

One of the strengths of this platform is the possibility of shared parameter fitting, also called global fitting. It means that large data sets are fitted simultaneously and some of the parameters are shared, e.g., reaction orders and rate constants. The final step is verifying the selected model through further experiments and fitting the obtained data with the selected model.

In this work, the possible models were selected based on negative scaling exponents of the datasets and the linear dependence of double logarithmic plots. The reaction orders for primary (*n*_c_) and secondary nucleation (*n*_2_) were fixed in all datasets. In all cases, *n*_c_ was set to 2, and n_2_ to 1 based on the assumption that the mechanism would be the same (or at least similar) as in our previous work (Hovanová et al., 2023). The rate constants (*k*_+_*k*_n_ and *k*_+_*k*_2_) in unseeded datasets and all three rate constants (*k*_n_, *k*_+_, *k*_2_) in seeded experiments were set as global (shared) parameters. The average fibril length of the seeds was determined to be 495 by analyzing TEM images and utilizing ImageJ software(Schneider et al., 2012). Taking into account an average protein density of 1.35 g/cm^3^ (Fischer et al., 2004), the aggregate number concentration, P_0_, was then computed using the formula M_0_/L (Meisl et al., 2016), with L representing the average fibril length. This method was chosen to reduce the number of variables in the fitting process.

The best-fitting results were obtained by the model of secondary nucleation, where the surfaces of fibrils catalyze the nucleation of new aggregates from monomeric peptides. The differential equations describing the system of secondary nucleation are [see SI-S10 and S11 in Meisl et al. (Meisl et al., 2016)]:

$\frac{dP}{dt}= k_{n}m({t)}^{n_{c}}+ k_{2}m({t)}^{n_{2}}+ M(t)$ (Equation 1)

$\frac{dM}{dt}= {2m(t)k}_{+}P(t)$ (Equation 2)

The selected model was validated by adding 0.5% seeds to the system, followed by a successful fitting of the obtained data. Every dataset was fitted 10 times resulting in similar values of rate constants with the same quality of fits. The mean value and standard deviations were calculated from the obtained values of parameters. The curves with the values of the parameters obtained in this way are shown in Figure 2.

*Curve fitting with the sigmoidal curve.* The sigmoidal curve (Equation 3) (Nielsen et al., 2001) was used to fit the kinetic data of mixtures of chaotropic salts with 150 mM Pi:

$y=y_{i}- m_{i}t+ \frac{y_{f} - m_{f}t}{1+e^{\frac{- t-t_{0}}{\tau}}}$ (Equation 3)

The initial elongation slope is described by the term of the equation: $y_{f} - m_{f}t$. The apparent rate constant *k*_+_ is given as 1/τ, where τ is the time constant (the width of the range where the *y* value changes the most significantly). This method does not describe the molecular events of aggregation but allows comparison of kinetic data of fibril formation (Humenik et al., 2014; Nielsen et al., 2001).

Supporting Figures


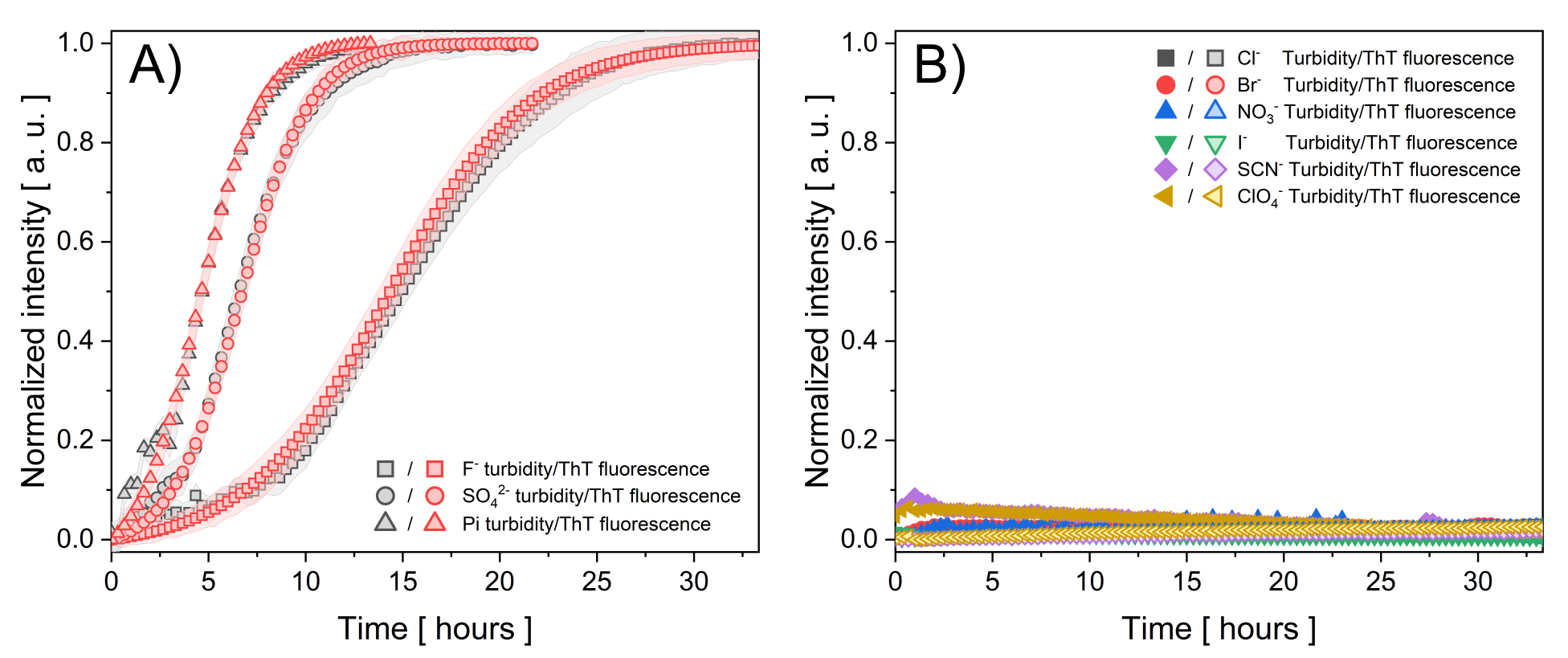


**Figure S1.** Kinetics of self-assembly of 15 μM protein eADF4(C16) in the presence of 150 mM kosmotropic (A) NaPi, Na_2_SO_4_, NaF, and (B) NaCl, NaNO_3_, NaBr, NaI, NaClO_4_, and NaSCN at 30 °C, were monitored using fluorescence of 30 μM ThT (ex. at 485 nm, em. at 528 nm) and simultaneously, change of turbidity over time at 340 nm. All data in (B) were normalized to the turbidity/ThT fluorescence maximum of Pi data in (A).

**
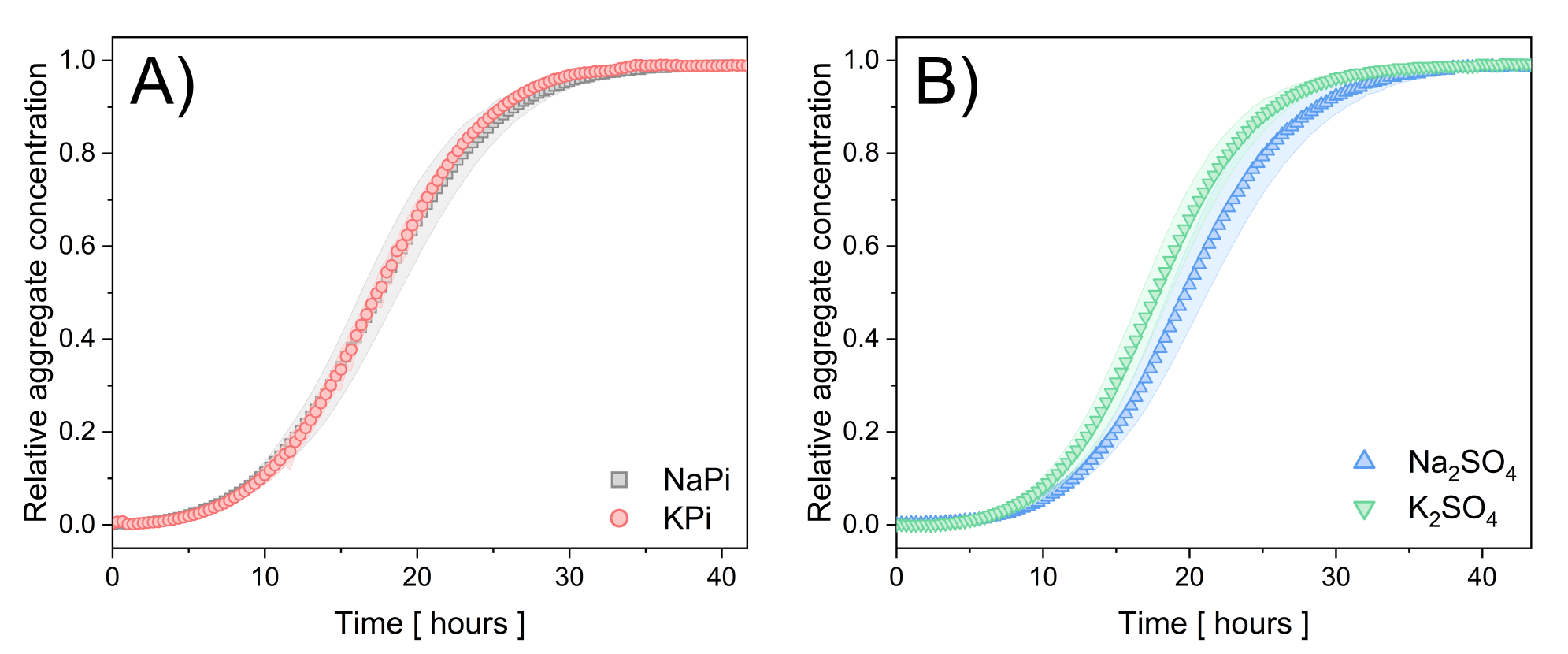
**

**Figure S2.** Self-assembly process evolution A) at 15 µM protein eADF4(C16) in the presence of 150 mM (**black**) NaPi, (**red**) KPi pH 8.0, at 20 °C, B) at 20 µM protein eADF4(C16) in the presence of 150 mM (**green**) K_2_SO_4_, (**blue**) Na_2_SO_4_, pH 8.0, at 20°C. The measurements were performed in 3 replicates for each condition. Sigmoidal curves represent a change of turbidity at 340 nm.


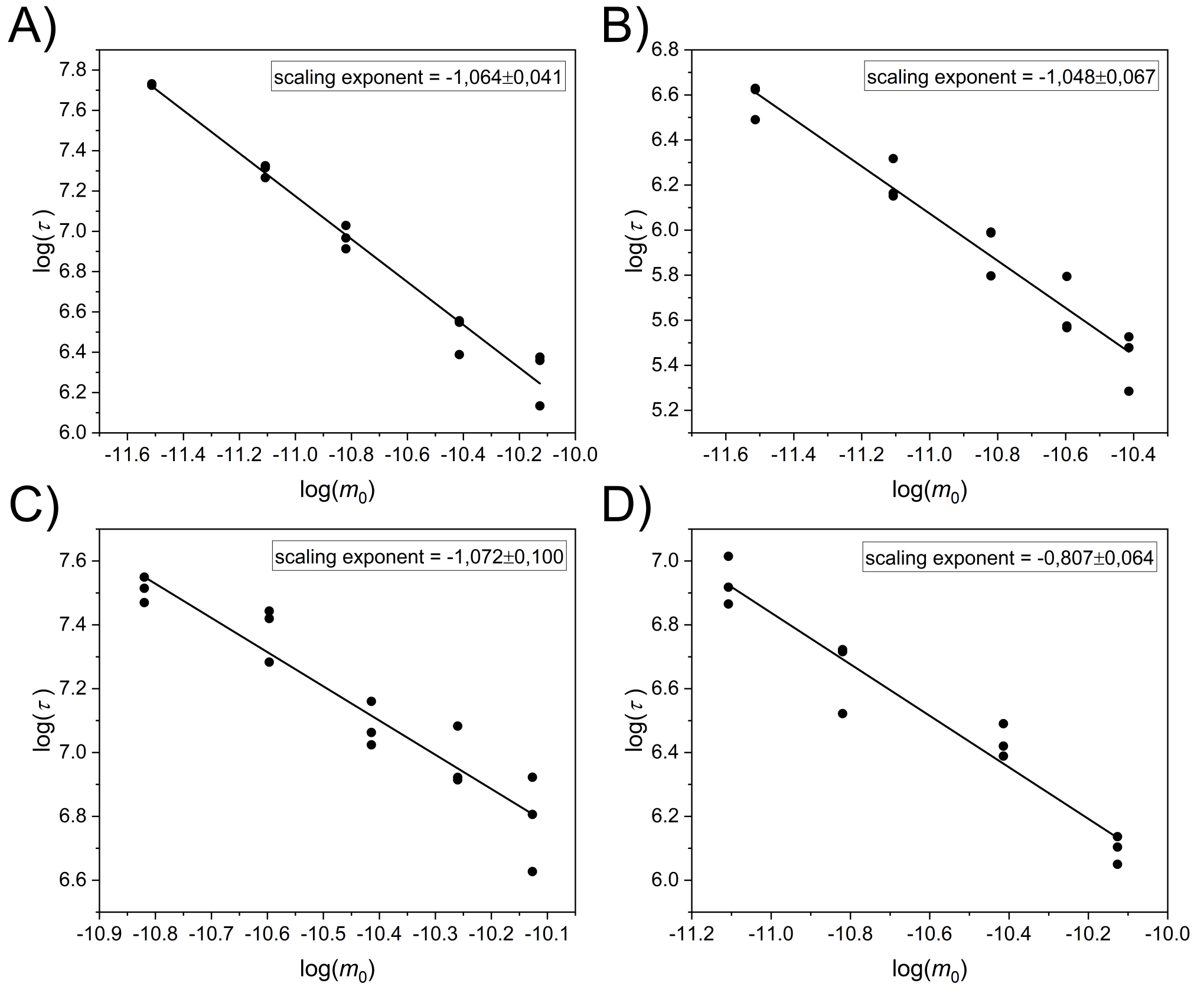


**Figure S3.** Plots with the double logarithmic scale of the half-time dependence on the corresponding protein concentration. The values for half-times are calculated from the sigmoidal curves in Figure 2. They represent aggregation kinetics of the protein eADF4(C16) with 150 mM A) Na_2_SO_4_ without seeds, B) Na_2_SO_4_ with 0.5% seeds, C) NaF without seeds, D) NaF with 0.5% seeds. Half-time plots and calculated scaling exponents were used as the first guide for the dominant aggregation mechanism. Power functions fit show that only one mechanism is dominant during the aggregation. Negative numbers of scaling exponents indicate that the half-times decrease as the monomer concentration increases.


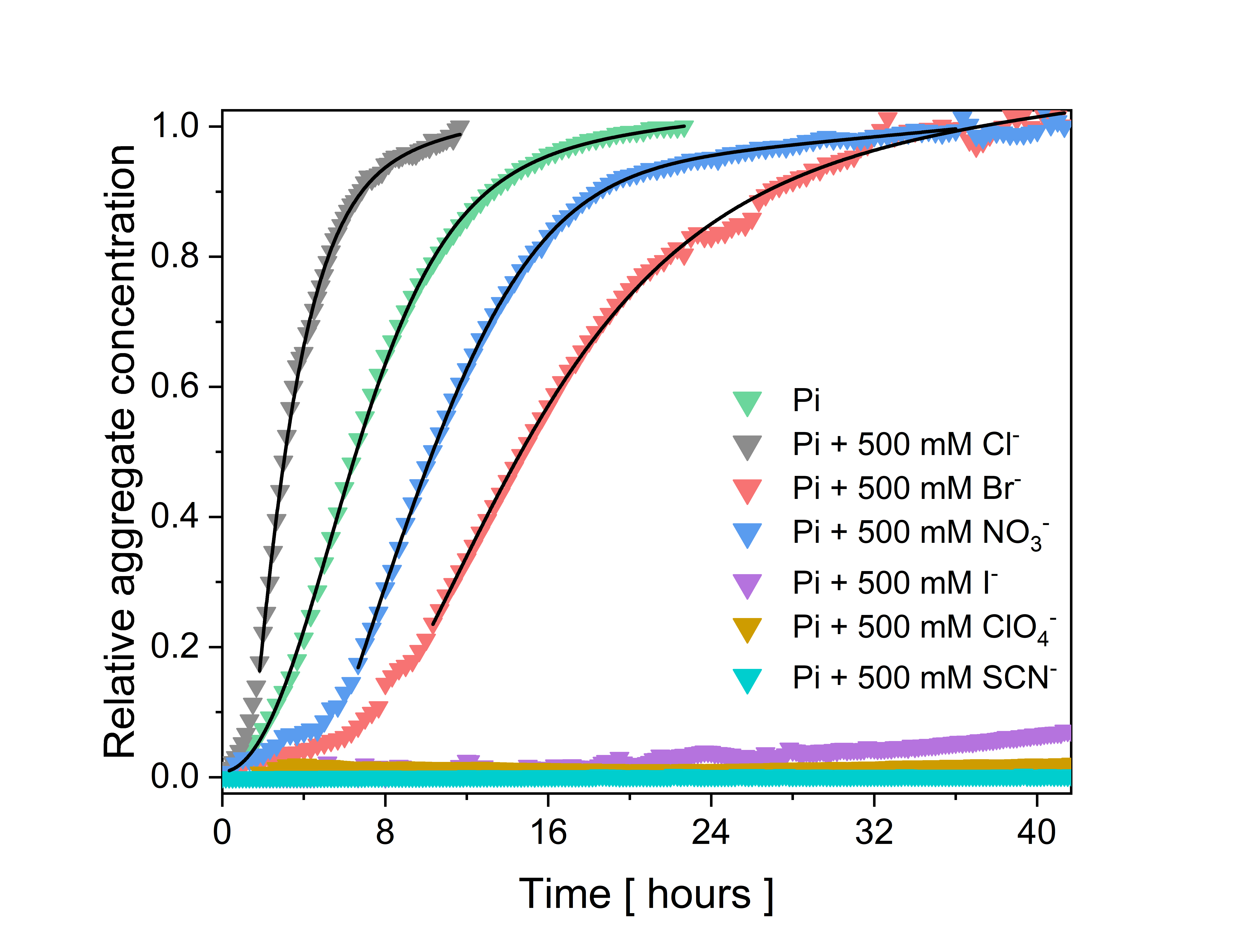


**Figure S4.** Kinetics of fibril formation under standard conditions at 150 mM KPi compared to fibril formation with the addition of 500 mM kosmotropic or chaotropic salt, at 30°C. The normalized sigmoidal curves represent the self-assembly of protein eADF4(C16), measured by the change of turbidity at 340 nm. The data are the average of five replicates for each condition. Curves were fitted by logistic function (Equation 3) (Nielsen et al., 2001).


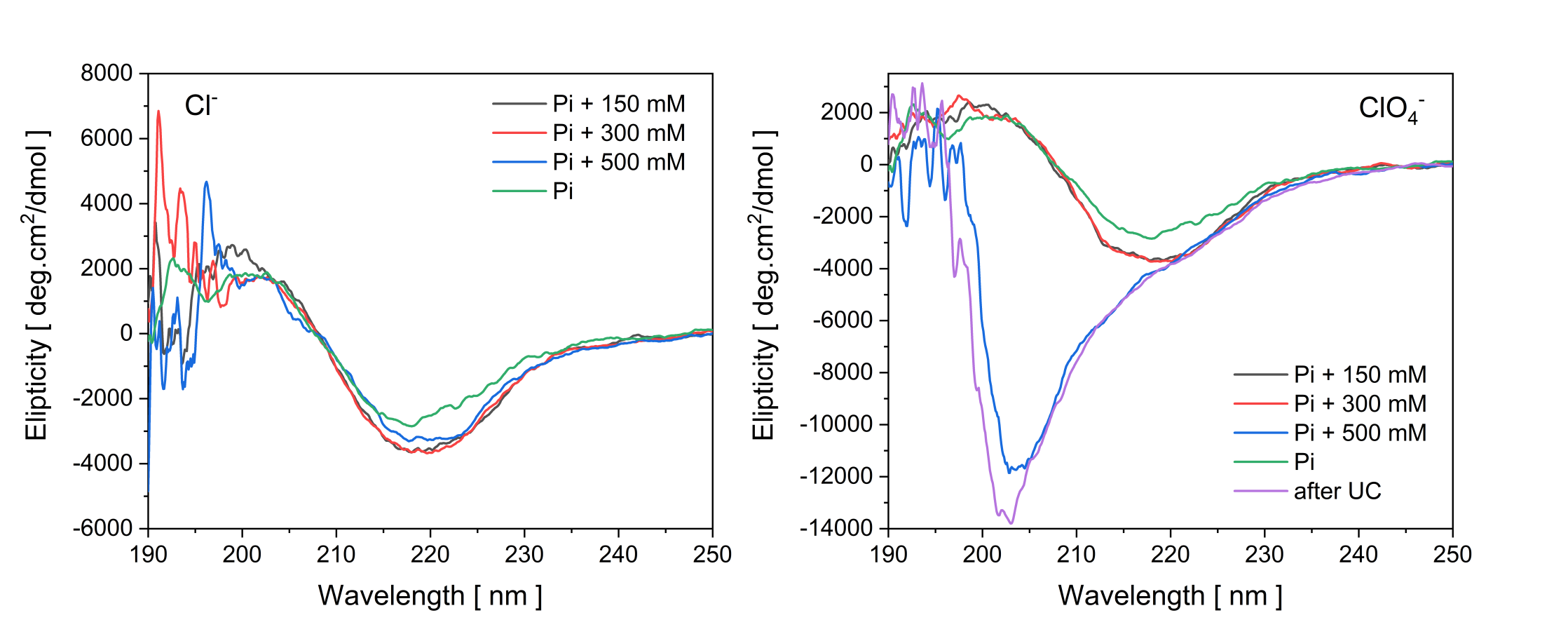


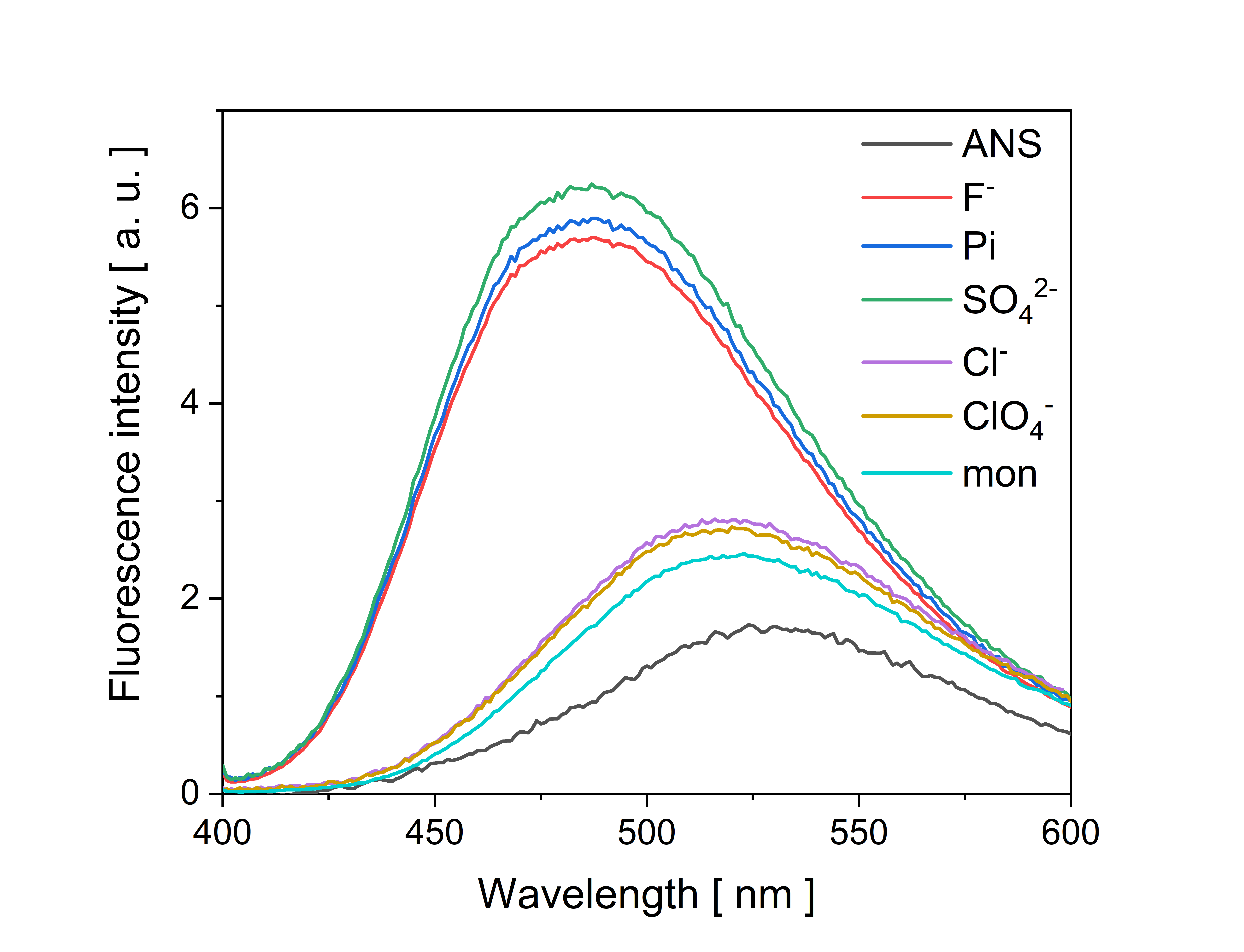
**Figure S5.** The far-UV CD spectra of eADF4(C16) after 48 hours of incubation in the presence of 150 mM Pi, used as control as well as with additional chloride (left panel) and perchlorate (right panel) at 150/300/500 mM salt. The purple line represents freshly dialyzed protein after ultracentrifugation (UC).

**Figure S6.** Fluorescence intensity of (black) 1 mM ANS, (turquoise) 1mM ANS with the 10 μM protein eADF4(C16) after the ultracentrifugation in monomeric state, and 1mM ANS with 10 μM protein eADF4(C16) that was incubated in (blue) NaPi, (green) Na_2_SO_4_, (red) NaF, (purple) NaCl, and (brown) NaClO_4_ for 48 hours. The emission spectra were measured upon excitation at 355 nm.

Supporting Table

**Table S1.** Corresponding rate constants of kinetic measurements showed in Figures 4A-D, for 3 different salt concentrations in the presence of 150 mM KPi and 15 μM eADF4(C16).

|  | k_+_ [s^-1^] | | | | | |
| --- | --- | --- | --- | --- | --- | --- |
| Salt concentration  [mM] | NaCl  + KPi | NaBr  + KPi | NaNO_3_  + KPi | NaI  + KPi | NaClO_4_  + KPi | NaSCN  + KPi |
| 0 | 0.366 | 0.366 | 0.366 | 0.366 | 0.366 | 0.366 |
| 150 | 0.548 | 0.284 | 0.310 | 0.264 | 0.216 | 0.098 |
| 300 | 0.593 | 0.243 | 0.265 | 0.131 | 0.194 | - |
| 500 | 0.600 | 0.192 | 0.253 | - | - | - |

Supporting References

Fischer, H., Polikarpov, I., & Craievich, A. F. (2004). Average protein density is a molecular-weight-dependent function. *Protein Science*, *13*(10), 2825-2828. <https://doi.org/10.1110/ps.04688204>

Hovanová, V., Hovan, A., Žoldák, G., Sedlák, E., & Humenik, M. (2023). Global analysis of kinetics reveals the role of secondary nucleation in recombinant spider silk self-assembly. *Protein Science*, *n/a*(n/a), e4722. <https://doi.org/https://doi.org/10.1002/pro.4722>

Humenik, M., Magdeburg, M., & Scheibel, T. (2014). Influence of repeat numbers on self-assembly rates of repetitive recombinant spider silk proteins. *Journal of Structural Biology*, *186*(3), 431-437. <https://doi.org/10.1016/j.jsb.2014.03.010>

Meisl, G., Kirkegaard, J. B., Arosio, P., Michaels, T. C. T., Vendruscolo, M., Dobson, C. M., . . . Knowles, T. P. J. (2016). Molecular mechanisms of protein aggregation from global fitting of kinetic models. *Nature Protocols*, *11*(2), 252-272. <https://doi.org/10.1038/nprot.2016.010>

Nielsen, L., Khurana, R., Coats, A., Frokjaer, S., Brange, J., Vyas, S., . . . Fink, A. L. (2001). Effect of environmental factors on the kinetics of insulin fibril formation: Elucidation of the molecular mechanism. *Biochemistry*, *40*(20), 6036-6046. <https://doi.org/10.1021/bi002555c>

Schneider, C. A., Rasband, W. S., & Eliceiri, K. W. (2012). NIH Image to ImageJ: 25 years of image analysis. *Nature Methods*, *9*(7), 671-675. <https://doi.org/10.1038/nmeth.2089>
